# Optimization of Parallel Artificial Liquid Membrane Extraction for the Determination of Over 50 Psychoactive Substances in Oral Fluid Through UHPLC–MS/MS

**DOI:** 10.1002/dta.3894

**Published:** 2025-04-10

**Authors:** Martina Croce, Camilla Montesano, Ilenia Bracaglia, Francesco Bartolini, Marcello Mascini, Dario Compagnone, Manuel Sergi

**Affiliations:** ^1^ Department of Public Health and Infectious Diseases Sapienza University of Rome Rome Italy; ^2^ Department of Chemistry Sapienza University of Rome Rome Italy; ^3^ Department of Bioscience and Technology for Food, Agriculture and Environment University of Teramo Teramo Italy

**Keywords:** full factorial design, liquid chromatography‐mass spectrometry, oral fluid, parallel assisted liquid membrane extraction, psychoactive substances

## Abstract

Over the past decade, there has been a diversification of the psychoactive substances available among drug users, resulting in the expansion of a dynamic market of synthetic molecules that are challenging for drug of abuse testing. Multiclass analytical methods are useful to deal with these new psychoactive substances (NPS), but sample preparation can be difficult and generate significant amounts of chemical waste. The aim of this work was the development of a high‐throughput microextraction method for the determination of 56 drugs belonging to different pharmacological classes in oral fluid (OF), including both traditional drugs and NPS. In the proposed workflow, the OF sample is cleaned‐up by parallel artificial liquid membrane extraction (PALME) and analyzed by liquid chromatography–tandem mass spectrometry (LC–MS/MS). Two hundred microliters of OF are mixed with 1800 μL of carbonate buffer 0.5 M (pH 12) and 0.4 g of sodium chloride and inserted into a donor plate; the acceptor plate embed a dodecylacetate‐supported liquid membrane and an acceptor solution composed of 50 μL formic acid 0.1% in H2O: MeOH, 80:20 (v/v); the whole assemblage is placed on an orbital shaker for 120 min for extraction. A full factorial design has been employed for extraction optimization to make it suitable for LC–MS/MS. The developed method is an example of green chemistry and may be used for screening and quantitative purposes, with limits of detection ranging from 0.01 to 1.5 ng mL^−1^ and optimal performance in term of precision and accuracy for 49 out of 56 drugs tested.

## Graphical abstract

This study is related to the development of a microextraction method for the determination of 56 psychoactive substances in oral fluid. Sample clean‐up is obtained by parallel artificial liquid membrane extraction and analyzed by liquid chromatography–tandem mass spectrometry. A full factorial design has been employed for extraction optimization.

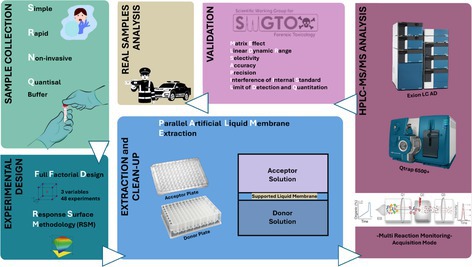

## 1 Introduction

Eighteen million years of “healthy” lives are lost every year because of drug use, and more than 35 million people worldwide suffer from drug use disorders [1]. Over the past 15 years, the market for psychotropic substances has substantially changed: from a relatively small number of illicit drugs, we assisted to a flood of a vast and heterogeneous set of substances active over the central nervous system, known as new psychoactive substances (NPS) [2]. These compounds, of natural and synthetic origin, mimic the effects of traditional drugs of abuse, posing a risk to public health due to the lack of pharmacological knowledge [1] or absent medicinal historical use [3]. Rapid spread of NPS across the public has been observed worldwide; the reasons underlying NPS use are both functional and circumstantial. On the one hand, subjective behavioral effects were found relevant, while on the other hand, the possibility of eluding established legal frameworks is appealing to the users [4].

The current clinical standard practice of drugs of abuse testing is based on immunoassay screening (IA) followed by the confirmation of positive findings with chromatographic techniques, such as gas and liquid chromatography (GC and LC) coupled with mass spectrometry (MS). For polydrug monitoring, the screening step is often problematic [5, 6], as methods are either not available or of low accuracy for several frequently used substances and, when available, often misunderstand which substances are present. With instrumentation improvements, there is an increasing trend toward developing comprehensive LC‐MS and GC‐MS methods, capable of detecting and quantifying multiple classes of drugs in a single assay.

Oral fluid (OF) is recognized as an important specimen for drug testing [7]; it can be used to confirm the recent consumption of all major types of abused drugs [8, 9, 10], including NPS [11, 12, 13]. Common applications of OF analysis are monitoring in substance abuse treatment programs, therapeutic drug monitoring, pain management, workplace drug testing, clinical toxicology, and driving under the influence of drugs (DUID) investigations. OF has a complex biochemical composition, containing various components like salts, organic small molecules, food debris, proteins, and lipids, which may cause a series of issues including LC column degradation, mass spectrometer contamination, signal interference, and most importantly, matrix effect (ME). This last phenomenon results in the enhancement or suppression of analyte ion intensity caused by coeluting matrix components [14]. However, due to the numerous factors affecting analyte extraction and matrix removal (e.g., choice of solvents, ion pairing agents, temperature, and buffer pH), the developing work for sample preparation can be difficult, tedious, and labor‐intensive, making it one of the most significant parts in the whole analytical method development [15]. Sample preparation mainly involves selective isolation of analytes of interest from the matrix, minimization or elimination of matrix components in processed samples and, if required, enrichment of analytes. Based on the analyte properties and matrix complexities, different sample preparation techniques have been developed for OF pretreatment, that is, protein precipitation (PPT), liquid–liquid extraction (LLE), and solid‐phase extraction (SPE) [16, 17, 18, 19, 20, 21, 22, 23]. These are the most traditional and commonly used techniques [24], but they generally need a moderate volume of sample and organic solvents. To account for these drawbacks, innovative miniaturized techniques such as single drop microextraction (SDME) [25], dispersive liquid–liquid microextraction (dLLME), hollow fiber–liquid‐phase microextraction (HF‐LPME) [26], and more recently, microextraction on packed sorbent (MEPS) [27] have been proposed. Among the LLME techniques, parallel artificial liquid membrane extraction (PALME) represents an innovative and valuable strategy for sample clean‐up; the technique is based on a three‐phase system, exploiting a pH gradient to selectively transfer basic/acidic analytes from the original matrix to an acidic/basic aqueous extracting phase with an intermediate passage into an organic solvent (2–3 μL) immobilized within the pores of a supported liquid membrane (SLM), usually in a 96‐well plate configuration.

To the best of our knowledge, the use of PALME has never been reported to date for OF pretreatment in drug testing. PALME was firstly proposed in 2013 by Gjelstad et al. [28] for the extraction of pethidine, nortriptyline, methadone, and haloperidol in plasma. More recent publications include other pharmaceuticals [29, 30, 31, 32, 33], biomarkers [34], and NPS belonging to the piperazine, phenetylamine, cathinone [35], and benzodiazepine classes [36] in plasma, whole blood, and dried blood spot [37] as well as fentanyl analogs in urine [38].

The aim of this study was the development of a novel method for multiclass drug testing in OF, exploiting PALME extraction and UHPLC‐MS/MS analysis; both traditional drugs and several NPS were considered. The advantages of using OF for toxicological applications are well‐known, and moderate volumes are normally available, making possible a significant enrichment of analytes through PALME. Giving the high number of included analytes and the multiple variables which can affect extraction rate when using PALME, optimization of extraction parameters was achieved through multiresponse analysis and experimental design, allowing to maximize the overall recoveries for the 56 analytes under investigation. The developed method was then validated according to ANSI/ASB Standard 036 guidelines and applied to some real OF samples collected during roadside drug screening services.

## 2 Experimental

### 2.1 Chemicals

Analytical standards of amphetamine, methamphetamine, 3,4‐methylenedioxy methamphetamine (MDMA), 3,4‐methylenedioxyamphetamine (MDA), cocaine, codeine, benzoylecgonine, norcocaine, morphine, 6‐monoacetylmorphine (6MAM), buprenorphine, norbuprenorphine, methoxetamine, methadone, ketamine, phencyclidine (PCP), 3,4‐methylenedioxy‐N‐ethylamphetamine (MDEA), 4‐iodo‐2,5‐dimethoxy‐N‐(2‐methoxybenzyl)phenethylamine (25‐I‐NBOMe), 4‐bromo‐2,5‐dimethoxyphenethylamine (2C‐B), 2,5‐dimethoxy‐4‐isopropylthiophenethylamine (2C‐T‐4), 2,5‐dimethoxy‐4‐(propylsulfanyl)phenethylamine (2C‐T‐7), 2,5‐dimethoxyphenethylamine (2CH), 1‐(2‐fluorophenyl)‐2‐(methylamino)propan‐1‐one (2‐FMC), 2‐methoxymethcathinone (MeOMetCat), 4‐methyl ethil cathinone (4‐MEC), buphedrone, butylone, diethylcathinone, mephedrone, methylone, 3,4‐methylenedioxypyrovalerone (MDPV), α‐pyrrolidinopentiophenone (alpha‐PVP), n([1‐[(1‐methylpiperidin‐2‐yl)methyl]‐1H‐indol‐3‐yl](2,2,3,3‐tetramethylcyclopropyl)‐methanone (AB005), [(11R)‐2‐methyl‐11‐(morpholin‐4‐ylmethyl)‐9‐oxa‐1‐azatricyclo[6.3.1.04,12]dodeca‐2,4(12),5,7‐tetraen‐3‐yl]‐naphthalen‐1‐ylmethanone (WIN55,212), (1‐(2‐morpholin‐4‐ylethyl)indol‐3‐yl)‐naphthalen‐1‐ylmethanone (JWH200), and hydrochloride salts of acetylfentanyl, acrylfentanyl, alfentanyl, butyrfentanyl, fentanyl, furanylfentanyl, ocfentanil, norfentanyl, fluorofentanyl, remifentanil, α‐ methyl fentanyl, α‐methyl thiofentanyl, 𝛽‐hydroxy fentanyl, (±)‐*cis*‐3‐methyl fentanyl, (±)‐*cis*‐3‐methyl thiofentanyl, as well as, sufentanyl citrate were purchased from Cayman Chemical (Ann Arbor, Michigan, United States) in the form of methanolic solutions at 100 𝜇g mL^−1^. Stable isotopically labeled internal standards (ISs) including fentanyl‐d_5_, norfentanyl‐d_5_, ketamine‐d_4_, cocaine‐d_3_, methadone‐d_3_, Buprenorphine‐d_4_, mephedrone‐d_3_, 25‐C‐NBOMe‐d_3_, MDMA‐d_5_, MDPV‐d_8_, amphetamine‐d_6_ and were obtained from the same supplier as methanolic solutions at 100 𝜇g mL^−1^. Standards of lormetazepam, triazolam, alprazolam, clonazepam, oxazepam and labeled ISs alprazolam‐d_5_ and diazepam‐d_3_ were purchased from Lipomed (Arlesheim, Switzerland) as 100 𝜇g mL^− 1^ methanolic solutions. Working solutions at 1 𝜇g mL^−1^ were prepared for each analyte in methanol by diluting the original stock solution and were kept at −20°C. A mixture of all the analytes was also prepared at the same concentration.

### 2.2 Sample Collection

OF was collected with a Quantisal device (Abbott, Sesto San Giovanni, Milan). For method development, OF samples obtained from several laboratory workers were combined and mixed with three parts of Quantisal buffer.

Real samples were obtained from roadside drug enforcement services. Randomly selected drivers were initially tested through rapid immunoassay testing for cannabis, opiates, cocaine, amphetamine, methamphetamine, MDMA, benzodiazepines, and ketamine. The drivers, who resulted positive to the screening test, were asked to provide an additional OF sample for confirmatory analysis through UHPLC‐MS/MS analysis; this OF sample was collected with Quantisal device. Left‐over samples were transferred to our laboratory, and 200 μL of each sample were processed according to the procedure described in the following paragraph.

### 2.3 PALME Extraction

Extraction was carried out by means of a 96‐well MultiScreen‐IP filter plate with polyvinylidene fluoride (PVDF) porous membranes (0.45 mm pore size and 100 𝜇m thickness) (Merck KGaA, Darmstadt, Germany); each well included the SLM in contact with the acceptor solution. The donor plate consisted of a 96‐well polypropylene plate with 2.2 mL wells from Brand GMBH (Wertheim, Germany) and was clamped together with the acceptor plate for extraction. PALME involves a few simple passages; briefly, 200 μL of OF was mixed with 1800 μL of carbonate buffer 0.5 M (pH 12) containing ISs at 10 ng mL^− 1^ and inserted into the donor plate. 0.4 g of sodium chloride was added at this stage, and homogenization was assured by vortex mixing for 15 s. Afterwards, the acceptor plate, whose membrane was previously washed with ethanol and water as suggested by the purchaser guidelines, was placed upon the donor plate, and 3 μL of dodecylacetate containing 5% of trioctylamine was pipetted into the porous membrane to form the SLM. The acceptor solution, constituted by 50 uL of formic acid 0.1% in H2O: MeOH, 80:20 (v/v), was then added. Finally, the acceptor plate was sealed; for extraction, the whole assemblage was placed on an orbital shaker at 800 rpm for 120 min at room temperature. The acceptor solution was finally injected into the UHPLC–MS/MS system.

### 2.4 Extraction Optimization Through Multiresponse Analysis and Experimental Design

Data management and all statistical procedures were carried out using R software [39]. To investigate the extraction of illegal drugs, a comprehensive full factorial design was employed, incorporating three key factors: pH, solvent extraction, and methanol percentage in the acceptor solution. The chosen levels for each factor were as follows: pH at levels 9, 10, 11, and 12; dodecyl acetate (DoA), dihexyl ether (DiE), and decanol (DeC) as extraction solvents; methanol in the acceptor solution at the concentration percentage of 0, 10, 20, and 30. Forty‐eight experiments involving different conditions for the three key factors were conducted in duplicate (Table S1). The multiple response dataset was represented by the 56 drugs area obtained from LC‐MS/MS analysis. Each response was scaled using min‐max transformation. The RSM package [40] was employed to construct models for each of the 56 responses. This involved employing the main effects of the factors, as well as their interactions, to build models incorporating first order (FO), two‐way interaction (+TWI), and pure quadratic (PQ) terms. The models are aimed at capturing the intricate relationships between factors and responses, enabling the prediction of extraction outcomes under varying conditions. Then, to assess model prediction capabilities of the experimental design, the function “predict,” from the “stats” package, was applied [41]. Under the desirability package [42], we use the dTarget function with the following parameters: min = 0, target = 0.1, and max = 1. The desirability function method (by using the “optim” function from the “stats” package) was then carried out to obtain the global optimum across all 56 variables. The 3D surface plots were generated using the “persp,” function from the “graphics” package, illustrating the prediction of the 56 response values simultaneously. The plot showcased the optimal conditions within the real range for each solvent extraction (pH 9–12; methanol 0–30), enabling a visual representation of the interplay between factors and their impact on extraction outcomes. For each solvent extraction, the global desirability within the experimental region was visualized using the contour (“graphics” package) plot. The workflow was automated using a R script written as rmarkdown file and reported in the supporting information (File S1).

### 2.5 UHPLC–MS/MS Analysis

LC was performed on an Exion LC AD system equipped with a vacuum degasser, a temperature control device and an autosampler with a 20 uL loop (AB‐Sciex). A SCIEX 6500 QTRAP from AB‐Sciex (Toronto, Ontario, Canada) with a Turbo V ESI source and IonDriveTM high energy detector was used for MS detection. Analytes were separated using a Kinetex Polar C18 column (10 cm × 2.1 mm ID) from Phenomenex (Torrance, California, United States) packed with core–shell particles of 2.6 μm which was kept at 40°. Acetonitrile:methanol, 50:50 (*v*:*v*) + 0.1% formic acid (B) and water + 0.1% formic acid (A), were used as mobile phases at a flow rate of 0.4 mL min^−1^. The multistep gradient scheme for the separation was as follows: Phase B was kept at 0.5% for 1.2 min and then increased to 10% in 1.8 min, to 20% in 1 min, to 30% in 1 min, to 33% in 1 min, to 45% in 1 min, to 50% in 1 min, to 65% in 2 min, to 85% in 1 min, and finally, to 100% in 1.5 min. These conditions were held for 2 min, and re‐equilibration to the initial conditions was achieved in 2.5 min. The total run time was 18.5 min.

**FIGURE 1:**
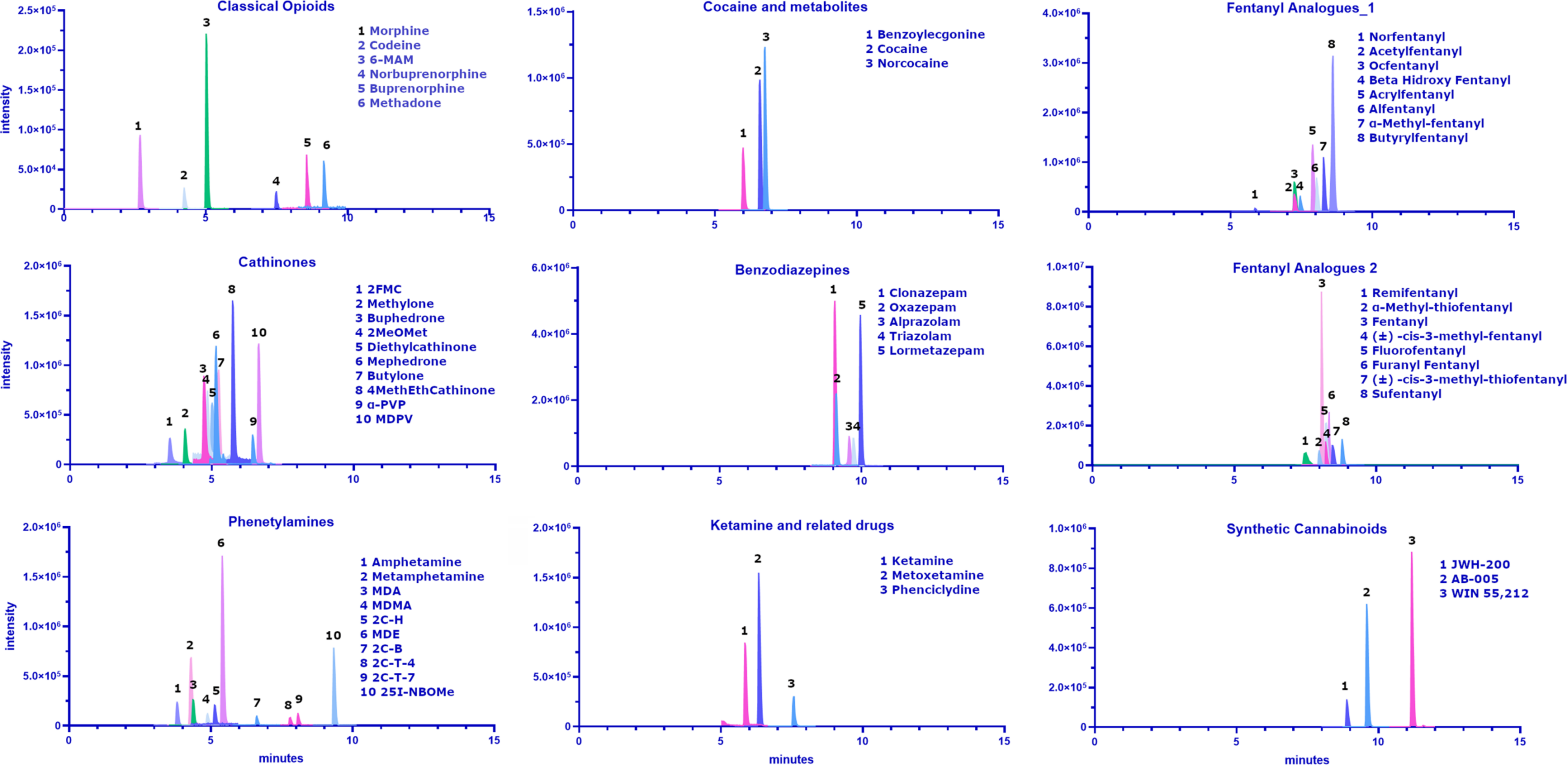
Representative chromatogram of a drug‐free oral fluid sample spiked with a mixture of analytes at 5 ng ml^−1^. The analytes are grouped based on the chemical class.

All the analytes were detected in positive ionization, with a capillary voltage of 4500 V, nebulizer gas (air) at 55 psi, turbo gas (nitrogen) at 60 psi, curtain gas at 30 psi, and collision gas medium; the source temperature was 450°C. For each analyte, two multiple reaction monitoring (MRM) transitions were selected. All source and instrument parameters for the monitored analytes were tuned by injecting each single standard solution at a concentration of 10 ng mL^−1^ at 10 μL min ^−1^ by a syringe pump. All the source parameters have been checked in flow injection analysis with the same chromatographic conditions. Data visualization was performed with AB‐Sciex software, while integration and data analysis were carried out using AB‐Sciex software SCIEX OS. The selected transitions, together with the main LC–MS/MS parameters, are reported in Table 1, while a representative chromatogram of a OF sample spiked at 10 ng mL^−1^ is reported in Figure 1.

**TABLE 1:** Retention times and MRM acquisition parameters for all analytes and deuterated internal standards (in bold).

Analyte	Rt (min)	Precursor ion (m/z)	DP (V)	EP (V)	Product ions (m/z)	CE (V)	CXP (V)
**25C‐NBoMe D3**	8.8	339.1	31	10	124.1	27	18
25I‐NBoMe	9.36	428	81	8	121.1	25	10
					91	75	12
2C‐B	6.5	260.4	36	10	228.1	27	12
					243.2	17	14
2C‐H	5.17	182.1	12	8	165.1	15	10
					150	29	8
2C‐T‐4	7.83	256.3	40	13	167.1	39	15
					91.1	58	10
2C‐T‐7	8.08	256.3	40	13	167.1	39	15
					91.1	58	10
2‐FMC	3.6	182.2	18	9	164.1	21	26
					149	24	29
2‐MeOMet‐Cathinone	4.9	194.1	50	8	176.3	17	15
					161.1	25	13
4‐MethEt‐Cathinone	5.77	192.1	11	12	174.1	19	28
					145	28	21
6‐MAM	5.02	328.2	134	3	165.3	48	14
					180.9	51	22
AB005	9.62	353.2	96	10	112.1	30	9
					98.1	30	10
Acetylfentanyl	7.28	323.2	37	6	188.5	29	6
					105.2	42	12
Acrylfentanyl	7.92	335.2	124	11	188.3	29	11
					105.1	39	16
Alfentanyl	8.07	417.2	54	5	197.1	36	18
					165.4	44	17
α‐Methyl‐fentanyl	8.3	136.0	6	10	91	65	14
					119.2	38	18
α‐Methyl‐thiofentanyl	8.02	290.1	52	6	125.1	30	11
					97.2	27	21
α‐PVP	6.4	353.4	71	10	91	71	12
					77.1	31	12
**Alprazolam D5**	9.6	314.3	43	6	286.3	42	34
Alprazolam	9.6	178	75	11	205.2	56	8
					281.2	35	13
**Amphetamine D6**	3.8	142	6	10	93	21	14
Amphetamine	3.8	136	6	10	99	21	14
					119.1	11	14
Benzoylecgonine	5.0	290.1	52	6	168.1	20	15
					105	25	9
β‐Hydroxy‐fentanyl	7.5	353.2	67	5	132.1	43	15
					186.2	33	20
Buphedrone	4.72	178.2	12	10	160.3	17	24
					132.1	24	15
**Buprenorphine D4**	8.6	472	85	10	59.1	100	7
Buprenorphine	8.6	357.3	142	8	414.3	47	21
					396.1	53	40
Butylone	5.3	222.1	22	10	174.2	25	24
					204.1	17	17
Butyrylfentanyl	9.6	351.4	76	6	188.2	31	15
					105.2	43	12
(±)‐*Cis*‐3‐methyl‐fentanyl	8.5	351.2	174	11	202.1	28	22
					105.1	39	16
(±)‐*Cis*‐3‐methyl‐thiofentanyl	8.35	357.3	86	8	208.1	31	18
					111.1	40	13
Clonazepam	9.1	316.3	25	8	270.2	35	42
					240.9	47	34
**Cocaine D3**	**6.6**	**307**	**26**	**9.5**	**184.9**	**26**	**5**
Cocaine	6.6	304	50	5	182.0	20	15
					82.0	30	15
Codeine	4.2	300.8	81	5.5	166	52	20
					153.2	72	12
Diethylproprion	5.1	206.1	51	10	105.1	29	12
					100.1	29	12
**Fentanyl D5**	8.1	342.4	52	12	105.2	51	15
Fentanyl	8.1	337.2	85	8	188.1	33	15
					105.0	43	11
Fluorofentanyl	8.3	355.4	25	6	188.1	34	21
					105.2	50	12
Furanylfentanyl	8.4	375.2	113	7	105.2	59	12
					188.2	29	16
JWH200	8.9	385.1	116	10	114.1	31	10
					155.1	27	14
**Ketamine D4**	5.8	242.1	45	8	129.2	38	9
Ketamine	5.8	238.0	36	10	125.2	34	14
					207.2	20	18
Lormetazepam	10	335.0	80	6	289.3	32	13
					317.2	20	15
MDA	4.4	180.1	20	5	163.2	14	14
					133.0	24	19
MDEA	5.5	208.1	26	10	163.2	17	14
					105.0	33	12
**MDMA D5**	4.8	199.2	10	9.6	165.0	17	2
MDMA	4.8	194.1	56	10	163.3	15	22
					135	13	14
**MDPV D8**	6.7	284.3	26	9	134.3	37	38
MDPV	6.7	276.2	80	10	126.1	31	11
					135.2	32	12
**Mephedrone D3**	5.2	181.2	46	10	148.1	26	6
Mephedrone	5.2	178.2	26	5	160.3	16	13
					145.2	28	10
Methamphetamine	4.3	150.2	11	10	91.0	25	14
					119.3	13	12
**Methadone D3**	9.2	313.4	184	10	105.0	33	16
Methadone	9.2	310.4	184	10	105.0	33	16
					265.3	20	12
Methylone	4.0	208.2	11	10	160.2	24	11
					190.2	17	16
Methoxetamine	6.4	248.2	16	10	121.2	39	14
					203.2	15	20
Morphine	2.7	286.0	115	7	165.3	50	14
					152.4	76	12
Norbuprenorphine	7.5	414.3	178	9	165.4	92	13
					83.2	77	13
Norcocaine	6.7	290.0	61	10	168.2	21	14
					136.2	29	12
**Norfentanyl D5**	5.9	238.3	31	10	84	30	12
Norfentanyl	5.9	233.2	65	10	84.1	23	13
					150.3	23	12
Ocfentanyl	7.3	371.1	84	10	105.4	55	8
					188.3	22	12
Oxazepam	9.2	287.2	50	8	269.0	34	12
					241.1	21	14
Phencyclidine	7.6	244.2	20	10	159.1	15	10
					91.1	18	15
Remifentanyl	7.3	377.2	43	7	113.1	35	18
					146.3	36	12
Sufentanyl	8.8	387.2	54	9	238	26	23
					355.3	27	24
Triazolam	9.7	343.2	80	11	308.4	37	14
					315.4	39	15
WIN−55,212	11.2	427.1	121	10	155	33	16
					127.1	81	14

### 2.6 Validation

The optimized method was validated according to ANSI/ASB Standard 036 [43], by considering the following parameters: selectivity, linearity, recovery, precision, accuracy, ME, limits of detection (LODs), and limits of quantification (LOQs). Quality control (QC) samples were prepared in a pool of drug‐free OF/Quantisal, using samples obtained from different subjects; before QC preparation, OF drug‐free pool was analyzed, and the absence of any analyte signal was verified.

Selectivity was tested by analyzing different samples of blank OF and verifying the absence of analyte signal. The possible interference deriving from IS was assessed by adding the IS mixture to OF samples before extraction. To evaluate whether common pharmaceuticals and endogenous metabolites (paracetamol, methylphenidate, tryptophan, dopamine, lamotrigine, and caffeine) interfere with the assay, neat OF samples were fortified with the potential interferences at high concentrations and analyzed.

Carry‐over was evaluated by injecting blank samples before and after the highest calibrator sample for five times and verifying the absence of target analyte signal.

For each compound, two product ions were selected; positive identification was assessed through the MRM detection of both fragments at a retention time within 2% from that of a standard and with quantitative (Q) to qualitative (q) ion ratio within 20%.

To evaluate linearity, calibration curves were prepared in H2O: MeOH (80:20, *v*/*v*) containing 0.1% formic acid; seven calibrators at different concentration levels (*n* = 3) were prepared. Calibrators were analyzed in three different chromatographic runs and in three different working days. Linearity was evaluated from LOQ to 50 ng mL^−1^. Quantitation was performed by the internal standard method: Q/IS ratio was used to evaluate the calibration samples.

Dilution integrity was assessed by preparing OF samples spiked at 250, 500, and 1000 ng mL^−1^ (three samples for each concentration) and diluting 1:5, 1:10, and 1:20, respectively.

To evaluate LODs and LOQs, drug free‐OF samples from three different sources were spiked with a solution containing all the analytes at decreasing concentrations. All samples were processed and analyzed in duplicate over three separate runs

LODs were established as the lowest concentration of a drug that provided a signal‐to‐noise ratio (S/N) equal or greater than 3 for the lower MRM transition and fulfilled the identification criteria. LOQs were estimated likewise as the smallest concentration that gave a S/N ≥ 10. At LOQ, in addition to the verification of the identification criteria, relative standard deviation (RSD%) and accuracy were required to be within ± 20% (*n* = 3).

Precision and accuracy were calculated by using QCs samples at three concentration levels: LOQ value, 5 and 50 ng mL^−1^. Precision was calculated as the RSD%. Intraday precision (repeatability) was estimated for each analyte at each concentration, from the areas of five independent QCs spiked before extraction; interday precision (reproducibility) was studied by analyzing five QCs samples at each concentration on five different days. Accuracy was calculated in terms of bias, as the relative deviation (percent) of the mean concentration (*n* = 3), calculated by using a freshly prepared calibration curve, with respect to the corresponding spiked concentration. RSD% and accuracy were accepted at maximum values of 15% or 20% near LOQ.

Extraction recovery (R%) was determined at three different concentrations: LOQ value, 5 and 50 ng mL^−1^ by using five different OF samples. Each matrix source was spiked with an appropriate amount of standard solution and processed by PALME, while an identical number of blank samples were processed and spiked with the same amount of standard solution after the extraction step. Recovery was calculated as the ratio of the mean peak area of the samples fortified before PALME (*A*) and the mean peak area of the samples fortified after extraction (*B*). *R*% = *A*/*B* × 100.

To evaluate any potential interfering compounds included in the sample matrix, ME was calculated for each analyte by comparing the mean peak areas in solvent (C) with the mean peak area in post extraction fortified samples. Accordingly, ME% = B/C ×100. Variability of ME was evaluated by calculating at each concentration the RSD% of the calculated ME% values.

## 3 Results and Discussion

### 3.1 PALME

PALME was selected in this study for OF pretreatment, because it is characterized by a high‐throughput and low organic solvent consumption (few microliters), perfectly adhering to the principles of green chemistry [37].

The technique is particularly suitable for LC–MS/MS analysis because the aqueous extract is directly compatible with LC and high enrichment factors are possible with no issues in terms of ME.

PALME takes advantage of a SLM in a 96‐well plate configuration offering the possibility of processing several samples together; in the developed method, up to 96 samples can be processed in parallel in 120 min (1.25 min per sample). Extraction is based on a three‐phase system, exploiting a pH gradient to selectively transfer basic/acidic analytes from the original matrix to an acidic/basic aqueous extracting phase with an intermediate passage into an organic solvent immobilized within the pores of a membrane.

Because of the double step extraction (from the aqueous sample to the SLM and subsequently to the acceptor solution) and the need of establishing a pH gradient, which is the driving force for the extraction, the variables that influence the recovery rate of the analytes are multiple so that an appropriate design of experiments was deemed crucial for extraction step optimization.

#### 3.1.1 Evaluation of PALME Operational Parameters

The extraction of illegal drugs serves as an indispensable component of forensic analysis, providing critical insights into the composition and prevalence of illicit compounds within various matrices [44].

**FIGURE 2:**
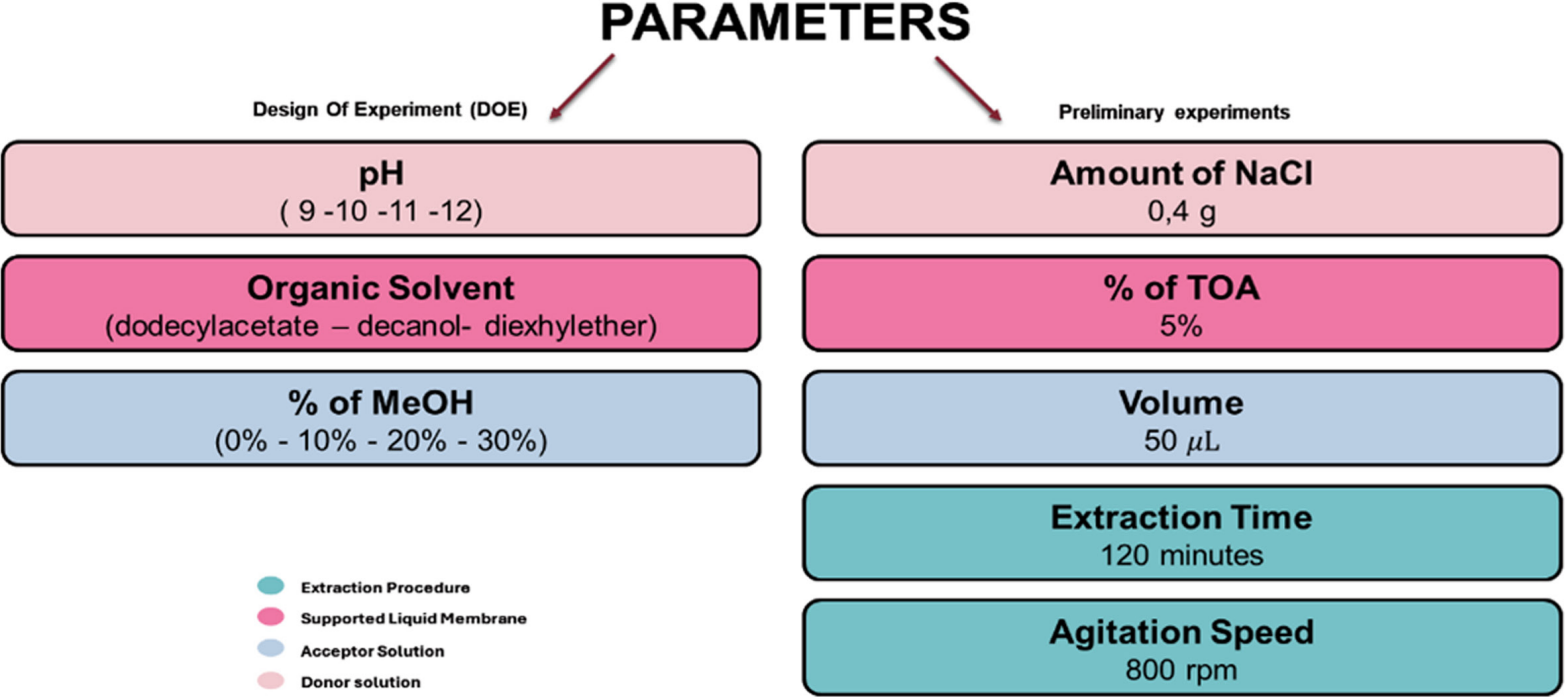
Schematic representation of the extraction parameters that were evaluated for PALME optimization.

The optimization of extraction conditions has emerged as a cornerstone in the pursuit of accurate and reproducible results. The potential for maximizing extraction yield and sensitivity holds profound implications for enhancing the sensitivity of downstream analytical methods, thereby enabling the detection of trace amounts of illicit substances [45]. This pursuit, however, is far from trivial, as extraction processes are inherently influenced by numerous interacting factors. To address this complexity, the incorporation of multiple response analysis and optimization techniques becomes indispensable. In this work, we used the synergy between full factorial design, response surface methodology (RSM) and multiple response optimizations to systematically explore parameter space and fine‐tuning conditions to achieve the best factors combination to perform the simultaneous extraction of 56 drugs [46]. This approach aids in navigating the trade‐offs between conflicting response variables by effectively addressing the challenge of multiple response resolution to increase analysis yield and sensitivity. Several parameters may influence the recoveries in PALME: the volume and pH of donor and acceptor solution, the ionic strength of the donor solution, the solvent employed in the SLM, the extraction time, and the composition of the acceptor solution (Figure 2).

For what concerns the volume of acceptor and donor solutions, the values were set to 50 μL and 2000 μL, respectively. The former was selected based on previous results that showed that 50 μL is the minimal employable volume, which guarantees high enrichment factors while providing reproducible results. On the other hand, the volume of donor solution must ensure a good contact between the membrane and the underneath solution and based on the volume of the used plates; 2000 μL was found to be optimal.

An initial pilot test was conducted to elucidate the impact of varying NaCl concentration and extraction time on the extraction process. Increasing NaCl concentration showed a positive correlation with the extraction of polar molecules, without compromising the efficiency of extraction for apolar ones. Consequently, the concentration of NaCl was set to its highest feasible values within the experimental framework. For what concerns extraction time, it was observed that for all the tested analytes, the extraction yield raised by increasing extraction time, but around 100 min of extraction, a plateau was reached, suggesting to set 120 min as optimal time. In order to simplify the following experimental design, the two discussed parameters were not further considered, and the values determined through one‐variable‐at time optimization were considered suitable, since a univocal response was observed for all the analytes.

#### 3.1.2 Design of Experiments

An in‐depth investigation regarding the optimal extraction conditions for all the 56 drugs was carried out by employing a comprehensive full factorial design that encompassed three essential factors: pH of the donor solution, extraction solvent, and acceptor solution composition. Given the basicity of most of the analytes, a pH gradient from basic to acidic pH was required to transfer the analytes from the matrix to the acceptor solution above the SLM. Analytes in OF must be in their protonated form so that a pH above the pKa value is essential. On the other hand, the composition of the acceptor phase can greatly influence the recovery; in fact, it was shown that the addition of small amounts of water‐soluble organic solvents, such as methanol, could be beneficial to improve analyte partitioning toward the acceptor phase, the optimal percentage of methanol, from 0% to 30%, into the acceptor phase was then investigated (larger amounts of methanol were not tested in order to avoid partial solubility of the acceptor solution with the organic solvent serving as SLM). Regarding the SLM, three solvents (i.e., DeC, DiE, and DoA) were tested. A suitable solvent must meet different requirements. First, it must have a good affinity with the analytes, and it must have a low volatility and be water immiscible.

**FIGURE 3:**
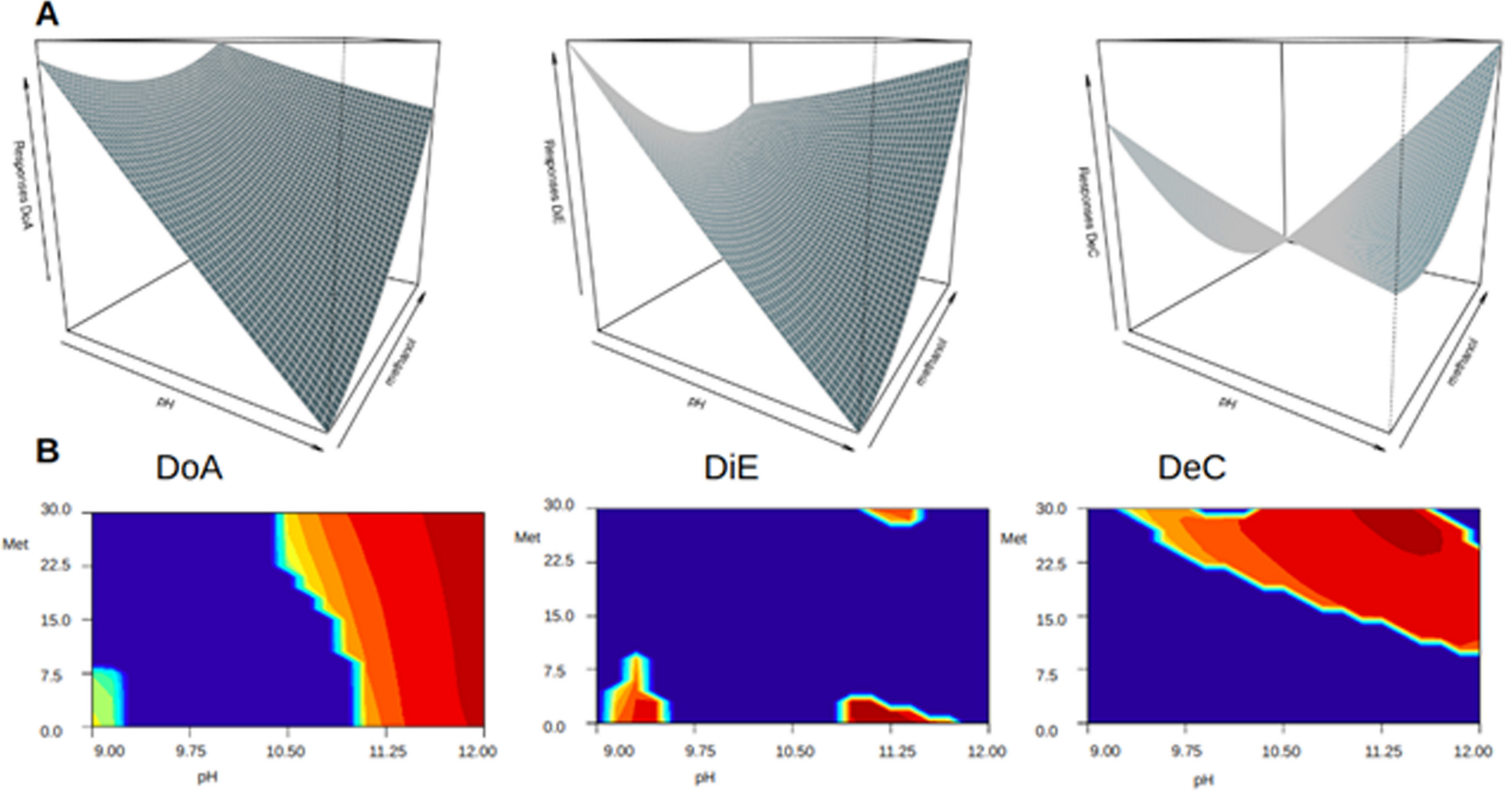
(A) 3D surface plots visually represented the prediction of all 56 response values simultaneously, within the real range for each solvent extraction method (pH 9–12 and methanol 0%–30%). (B) Contour plots for each solvent extraction, showing the global desirability within the experimental region of the pH–methanol parameters. DoA = dodecyl acetate; DiE = dihexyl ether; DeC = decanol.

The objective was to optimize the factor conditions across all drug extraction values using a multiple response analysis approach. In this work, we use the synergy between full factorial design, RSM, and multiple response optimizations to systematically explore parameters space and fine‐tuning conditions to achieve the best factors combination to perform the simulation extraction of 56 drug values simultaneously. This strategy consented to highlight the preferential conditions within the real range for each solvent extraction method (pH 9–12 and methanol 0%–30%) and provided a valuable visual representation of how the factors interacted and their impact on extraction outcomes. The plots reaffirmed the significance of pH, extraction solvent, and methanol concentration in the acceptor solution in influencing the extraction process and underscored the utility of the optimized conditions. The RSM function was used to build a predictive model for each of the three extraction solvents. The 3D surface plots reported in Figure 3 visually represented the prediction of all 56 response values simultaneously, highlighting the preferential conditions within the range for each solvent extraction method (pH 9–12 and methanol 0%–30%) and providing a valuable visual representation of how the factors interacted and their impact on extraction outcomes. The plots reaffirmed the significance of pH, extraction solvent, and methanol concentration in influencing the extraction process and underscored the utility of the optimized conditions.

The desirability approach was then employed to assess the overall desirability of the extraction conditions. This approach allowed us to evaluate and optimize the extraction conditions based on multiple response variables simultaneously. For each solvent extraction, the global desirability within the experimental region of the pH‐methanol parameters was visualized using contour plots (Figure 1B). These plots allowed for a comprehensive understanding of the desirability landscape and the regions where the extraction conditions met the specified desirability criteria. Upon optimizing extraction conditions, the best pH and methanol concentration for each solvent was reported in Table 2 along with global desirability value.

**TABLE 2:** Best pH and methanol concentration for each solvent along with global desirability value and the number of analytes below the target (0.1% of the max analytical signal) and below the detectable signal (0.01% of max analytical signal).

	Dodecyl acetate	Dihexyl ether	Decanol
Optimal pH	12.00	11.25	11.66
Optimal methanol %	20.60	0.35	29.5
Global desirability value	0.75	0.67	0.66

For DoA, the optimal pH was found to be 12. This pH level maximizes the desirability of the extraction process, indicating that for this solvent, higher pH conditions are favorable. DiE and DeC, had also an optimal response under basic conditions with an ideal pH of 11.25 and 11.66, respectively. These results suggested that for all solvents, elevated pH contributed to the simultaneous extraction of the drugs tested. High concentration of methanol was preferred for both DoA and DeC with optimal methanol percentage respectively of 20.6% and 29.5%. DiE, on the other hand, showed a significantly lower optimal methanol percentage of 0.3%. This solvent appears to benefit from an aqueous environment; probably higher concentrations of methanol in the acceptor solution result in a partial solubilization of the dihexylether layer and cause a decrease in the efficiency of the extraction.

The overall quality of the extraction process, considering multiple response variables simultaneously, was reported using the global desirability value. DoA showed the highest global desirability value at 0.75, suggesting that it was the most effective solvent among the three for this particular extraction.

DiE also exhibited a good global desirability value of 0.67, indicating its effectiveness in achieving desirable extraction outcomes. DeC had a global desirability value of 0.66, indicating a moderate performance; the number of analytes below the detectable signal was higher with this solvent suggesting that several substances may not be reliably quantifiable. The full factorial response data analyzed in the laboratory are shown as supporting information (Table S1). We could demonstrate that the real data agreed with the predicted data, showing that the three solvents had the best combination of pH‐methanol parameters in the predicted zones calculated by the global desirability optimization (Figure 3B, red zone).

These results highlighted the different performance of the three solvents under different pH‐methanol conditions and emphasized the importance of extraction parameters optimization in the simultaneous drug detection. DoA and DiE were both effective solvents, offering reliable quantification of a larger number of substances, while DeC showed lower performance in comparison.

The optimized extraction procedure was able to recover almost all the tested drugs which are a significant improvement over the standard extraction procedures. In conclusion, this study demonstrated that DoE can be used to optimize extraction procedures for drugs, leading to improved detection rates, more accurate results, and reduced costs in forensic analysis.

However, it is important to note that the results of this study may not be generalizable to all laboratories. The optimal conditions may vary depending on the specific equipment and reagents used. Therefore, it is important to validate the optimized extraction procedure in each laboratory before using it for forensic analysis.

### 3.2 Validation of the Optimized Method

Once the main parameters for PALME extraction were optimized, the whole method was validated; the results of the validation experiments are reported below.

The values for recovery and ME are shown in Table 3. It can be noticed that for most analytes, recovery values are higher than 50%; only seven analytes (benzoylecgonine, morphine, 6MAM, codeine, oxazepam, clonazepam, and WIN‐55,212) had a recovery lower than 5%, and for this reason, they were not included in the validation set. It is noteworthy that the low effectiveness of PALME in extracting these molecules could be expected because of their chemical characteristics: Benzoylecgonine and natural opioids are moderately polar, making these substances less suitable for LLE; for what concerns benzodiazepines, their low pK_a_ values may hinder mass transfer of analytes from the SLM into the aqueous acceptor solution. For these seven analytes, the developed method is only semi‐Q, as poor accuracy and precision were obtained because of the low recovery.

**TABLE 3:** Recovery and matrix effect values for the 56 target analytes.

Analyte	Recovery (%)	Matrix effect (%)
25I–NBOMe	87	−6
2C‐B	90	−7
2C‐H	56	−5
2C‐T‐4	99	−2
2C‐T‐7	99	−3
2‐FMC	42	−9
2‐MeOMet‐cathinone	51	−2
4‐MethEt‐cathinone	99	1
6‐MAM	< 5	6
AB005	74	3
Acetylfentanyl	99	−6
Acrylfentanyl	99	−1
Alfentanyl	98	−1
α‐Methyl‐fentanyl	95	−3
α‐Methyl‐thiofentanyl	97	1
α‐PVP	97	0
Alprazolam	34	−3
Amphetamine	85	−4
Benzoylecgonine	< 5	2
β‐Hydroxy‐fentanyl	98	−1
Buphedrone	99	−1
Buprenorphine	54	−7
Butylone	98	0
Butyrylfentanyl	99	−3
(±)*Cis*‐3‐methyl‐fentanyl	91	1
(±)*Cis*‐3‐methyl‐thiofentanyl	90	−1
Clonazepam	< 5	−3
Cocaine	65	1
Codeine	< 5	−8
Diethylproprion	98	0
Fentanyl	99	0
Fluorofentanyl	99	0
Furanylfentanyl	99	5
JWH200	22	6
Ketamine	99	4
Lormetazepam	7	−4
MDA	82	−2
MDEA	99	−1
MDMA	51	−2
MDPV	99	−3
Mephedrone	96	1
Methadone	83	−9
Methamphetamine	97	−2
Methylone	79	−1
Methoxetamine	96	5
Morphine	< 5	−13
Norbuprenorphine	40	0
Norcocaine	56	−2
Norfentanyl	55	−4
Ocfentanyl	98	−4
Oxazepam	< 5	−2
Phencyclidine	72	0
Remifentanyl	89	−2
Sufentanyl	94	−1
Triazolam	22	−5
WIN55,212	< 5	−23

ME was not significant for all the analytes, showing that PALME is an efficient clean‐up technique. This is an important feature of the method, as OF and, especially, swab collection buffers have been often reported to produce ion suppression or enhancement [47].

For what concerns LODs, the obtained values were between 0.01 and 1.5 ng mL^−1^, while LOQs were between 0.03 and 5 ng mL^−1^; the specific values for each analyte are reported in Table 4. Generally, these values are comparable to those reported in the literature [20, 48].

**TABLE 4:** Validation parameters, including precision, accuracy, LOD, and LOQ values.

Drug name	IS	Accuracy (%)			Intraday precision (CV%)			LOD (ng/mL^−1^)	LOQ (ng/mL^−1^)
L: 0.5 ng/mL^−1^; M: 5 ng/mL^−1^; H: 50 ng/mL^−1^		L	M	H	L	M	H		
25I–NBOMe	MDPV‐d8	87	84	116	11	19	5	0.02	0.06
2C‐B	MDMA‐ d5	88	110	119	11	5	7	0.30	0.90
2C‐H	MDMA‐ d5	96	118	102	17	17	1	0.40	1.30
2C‐T‐4	MDMA‐ d5	113	105	125	9	5	12	0.20	0.60
2C‐T‐7	MDMA‐ d5	107	106	118	15	2	10	0.30	0.90
2‐FMC	MDMA‐ d5	91	90	102	14	7	11	0.15	0.50
2‐MeOMet‐Cathinone	MDPV‐d8	94	87	89	17	11	4	0.40	1.25
4‐MethEt‐Cathinone	MDPV‐d8	116	112	119	6	7	4	0.05	0.20
AB005	Buprenorphine d4	119	113	112	10	10	4	0.02	0.06
Acetylfentanyl	Fentanyl‐d5	116	115	116	3	10	7	0.01	0.03
Acrylfentanyl	Fentanyl‐d5	115	115	114	6	8	7	0.01	0.03
Alfentanyl	Fentanyl‐d5	114	119	112	5	11	5	0.01	0.03
α‐Methyl‐fentanyl	Fentanyl‐d5	117	118	115	10	9	7	0.01	0.03
α‐Methyl‐thiofentanyl	Fentanyl‐d5	111	111	110	6	8	6	0.02	0.06
α‐PVP	MDPV‐d8	117	111	112	3	6	3	0.10	0.30
Alprazolam	Alprazolam‐d5	118	114	118	6	7	2	0.05	0.15
Amphetamine	Amphetamine‐d6	103	108	115	5	4	2	0.10	0.25
β‐Hydroxy‐fentanyl	Fentanyl‐d5	N/A	115	116	N/A	10	7	1.5	5.0
Buphedrone	MDPV‐d8	119	116	115	7	7	3	0.15	0.45
Buprenorphine	Buprenorphine‐d4	116	113	117	5	8	10	0.10	0.30
Butylone	MDPV‐d8	119	111	113	5	9	2	0.05	0.15
Butyrylfentanyl	Fentanyl‐d5	116	112	119	5	6	6	0.01	0.03
(±)*Cis*‐3‐methyl‐fentanyl	Fentanyl‐d5	119	117	113	8	6	9	0.01	0.03
(±)*Cis*‐3‐methyl‐thiofentanyl	Fentanyl‐d5	112	110	117	6	6	7	0.01	0.03
Cocaine	Cocaine‐d3	117	115	111	5	6	3	0.01	0.03
Diethylproprion	MDPV‐d8	117	116	113	8	2	4	0.03	0.09
Fentanyl	Fentanyl‐d5	115	114	102	6	9	1	0.01	0.03
Fluorofentanyl	Fentanyl‐d5	116	115	115	6	8	6	0.01	0.03
Furanylfentanyl	Fentanyl‐d5	110	119	118	5	9	6	0.01	0.03
JWH200	Methadone‐d3	99	87	85	3	7	1	0.05	0.15
Ketamine	Ketamine‐d4	118	110	119	5	7	4	0.10	0.30
Lormetazepam	Methadone‐d3	96	90	90	4	0	0	0.15	0.45
MDA	MDMA‐d5	91	99	109	11	13	1	0.05	0.15
MDEA	MDMA‐d5	116	117	116	5	6	7	0.02	0.06
MDMA	MDMA‐d5	83	86	81	14	12	6	0.20	0.60
MDPV	MDPV‐d8	115	117	115	6	7	4	0.03	0.09
Mephedrone	Mephedrone‐d3	114	82	112	5	4	4	0.10	0.40
Methadone	Methadone‐d3	93	82	112	14	7	8	0.15	0.45
Methamphetamine	MDMA‐d5	108	115	117	5	2	4	0.04	0.12
Methylone	Mephedrone‐d3	83	97	89	11	13	3	0.10	0.30
Methoxetamine	MDMA‐d5	119	117	119	6	6	4	0.01	0.03
Norbuprenorphine	Buprenorphine‐d4	N/A	90	95	N/A	18	18	1.0	3.0
Norcocaine	Cocaine‐d3	91	97	89	10	12	8	0.04	0.12
Norfentanyl	Norfentanyl‐d5	N/A	107	112	N/A	9	6	1.0	3.0
Ocfentanyl	Fentanyl‐d5	114	174	116	7	9	6	0.02	0.06
Phencyclidine	MDMA‐d5	116	119	112	5	8	10	0.04	0.12
Remifentanyl	Fentanyl‐d5	118	107	117	5	8	4	0.10	0.30
Sufentanyl	Fentanyl‐d5	117	118	119	9	7	6	0.01	0.03
Triazolam	Alprazolam‐d5	119	106	109	0	4	2	0.02	0.06

Calibration curves linearity was suitable for all the analytes in the tested range, between LOQ and 100 ng mL^−1^; all coefficients *R* ^2^ were higher than 0.99. The absence of carryover was assessed by injecting a blank after the injection of the higher calibration sample, and no signals above LOD were detected at the analyte retention time.

Intraday precision was evaluated by performing the analyses of QCs at three concentration levels (*n* = 5); good results were obtained with interday precision between 0% and 18%. Interday precision was evaluated by analyzing the same three QCs on three different working days; calculated RSD% was between 2% and 12% for all analytes. Results obtained for all analytes are reported in Table 4. Accuracy values are shown in the same table and were between 83% and 119%.

### 3.3 Method Comparison

Due to the multiple advantages of multianalyte methods in drug testing, several analytical protocols for simultaneously detect illicit drug belonging to different classes in OF have been proposed in the last years. In the present method, we included 56 traditional drugs and NPS that belonged to different chemical and toxicological classes with the purpose of developing a wide‐ranging analytical method; additional substances with similar structures can be added with minimal method development steps and comparable extraction rates would be expected. Only two studies encompassed a higher number of substances in OF [20, 49]; however, both methods were developed only for screening purposes, while the present method is suitable for quantitative analysis of 49 out of 56 drugs included in the method.

In addition, compared with other published methods, a clear advantage of PALME is the need for small volumes of organic solvent for sample preparation (13 μL per sample) with reduced MEs; to the best of our knowledge, no other method which includes an extraction step involves such a low consumption of organic solvent. Dilute‐and‐shoot approaches can totally avoid organic solvent consumption during sample preparation [50]; however, the drawbacks of this technique are well known and include suboptimal detection capability of certain analytes, increased probability of interferences and ME, and contamination of the LC‐MS instrument.

Another feature of the present method is related to the possibility of simultaneously extract 96 samples in 120 min resulting in an extraction time of 1.25 min per sample; similar extraction times were achieved by Valen et al. [51] by using a fully automated supported liquid extraction for the determination of 21 drugs.

Regarding the volume of OF used, it is generally lower or similar to other methods which typically report volumes of 90 uL [18] to 1000 uL [49].

### 3.4 Greenness of the Sample Preparation Method

The greenness of analytical procedures (GAC) is a multivariate, complex parameter that is not easily quantifiable [52]. The GAC approach originally excluded sample preparation from green analytical practices by suggesting in the first of its twelve principles to try avoiding this step [53]; green sample preparation (GSP) was recently defined and formulated in the form of 10 principles.

The greenness of the presented sample preparation method was evaluated by means of analytical GREEnness metric for sample preparation (AGREEEprep). The result is a pictogram indicating the final score, performance of the analytical procedure in each criterion, and weights assigned by the user [54]. The overall score is shown in the middle of the pictogram with values close to 1 and dark green color indicating that the assessed procedure is green. The performance of the procedure in each of the assessment criteria is reflected by the color in the segment with the number corresponding to each criterion.

**FIGURE 4:**
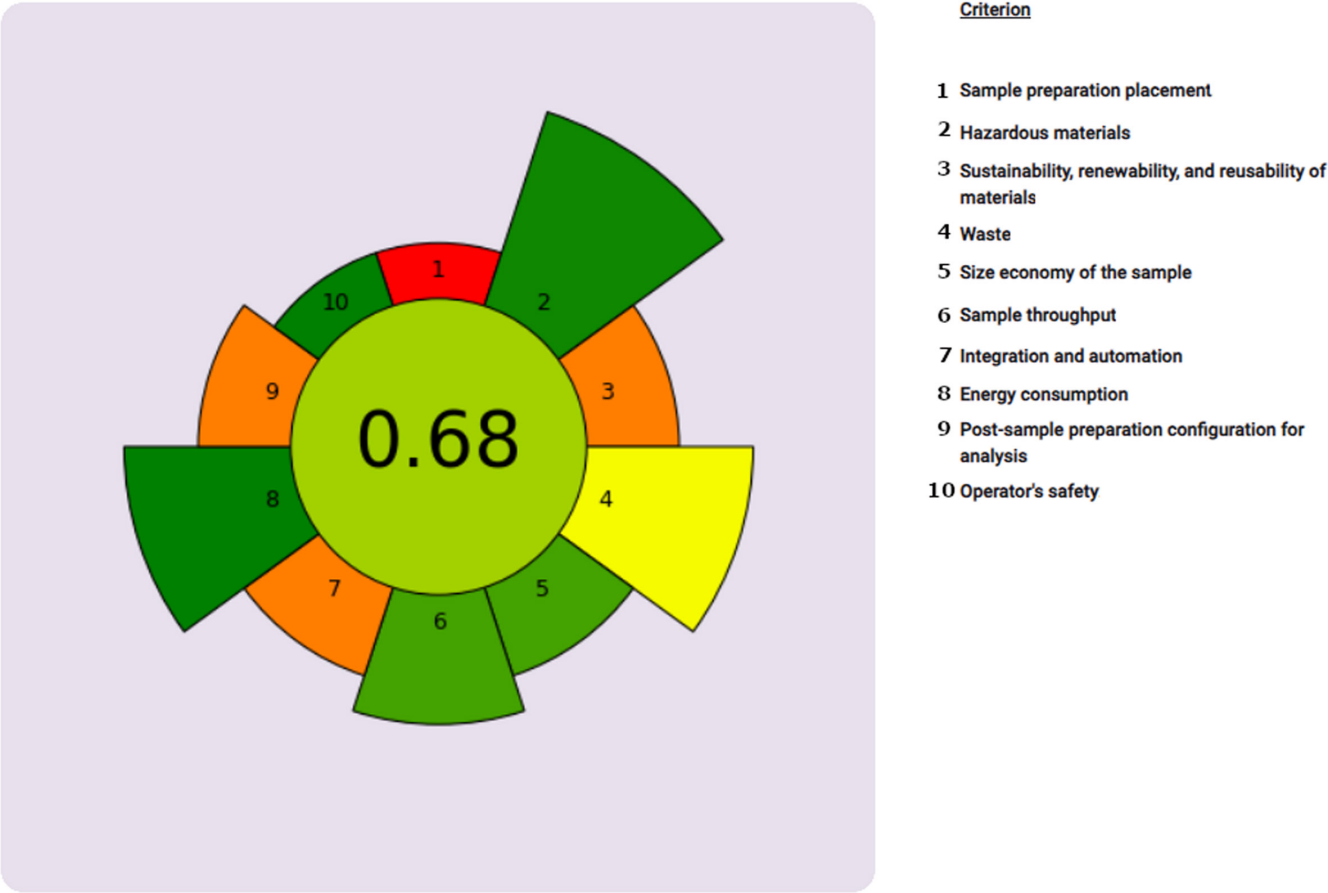
AgreePREP pictogram indicating the performance of the analytical procedure in term of greenness, according to several parameters and default weights. The overall score is shown in the middle of the pictogram.

By using default weight values for the 10 criteria, the AGREEprep calculator yielded an overall score of 0.68 to the developed method (Figure 4). It is acknowledged that an excellent green method should achieve a score of 0.75 or higher, while a score below 0.50 indicates insufficiency in terms of ecofriendliness [55]. The achieved score demonstrates that the developed method is optimal from the greenness point of view even if not excellent; the lower scores were obtained because of the use of LC‐MS postsample preparation and the limited reusability and sustainability of the materials used in PALME.

### 3.5 Real Sample Analysis

**FIGURE 5:**
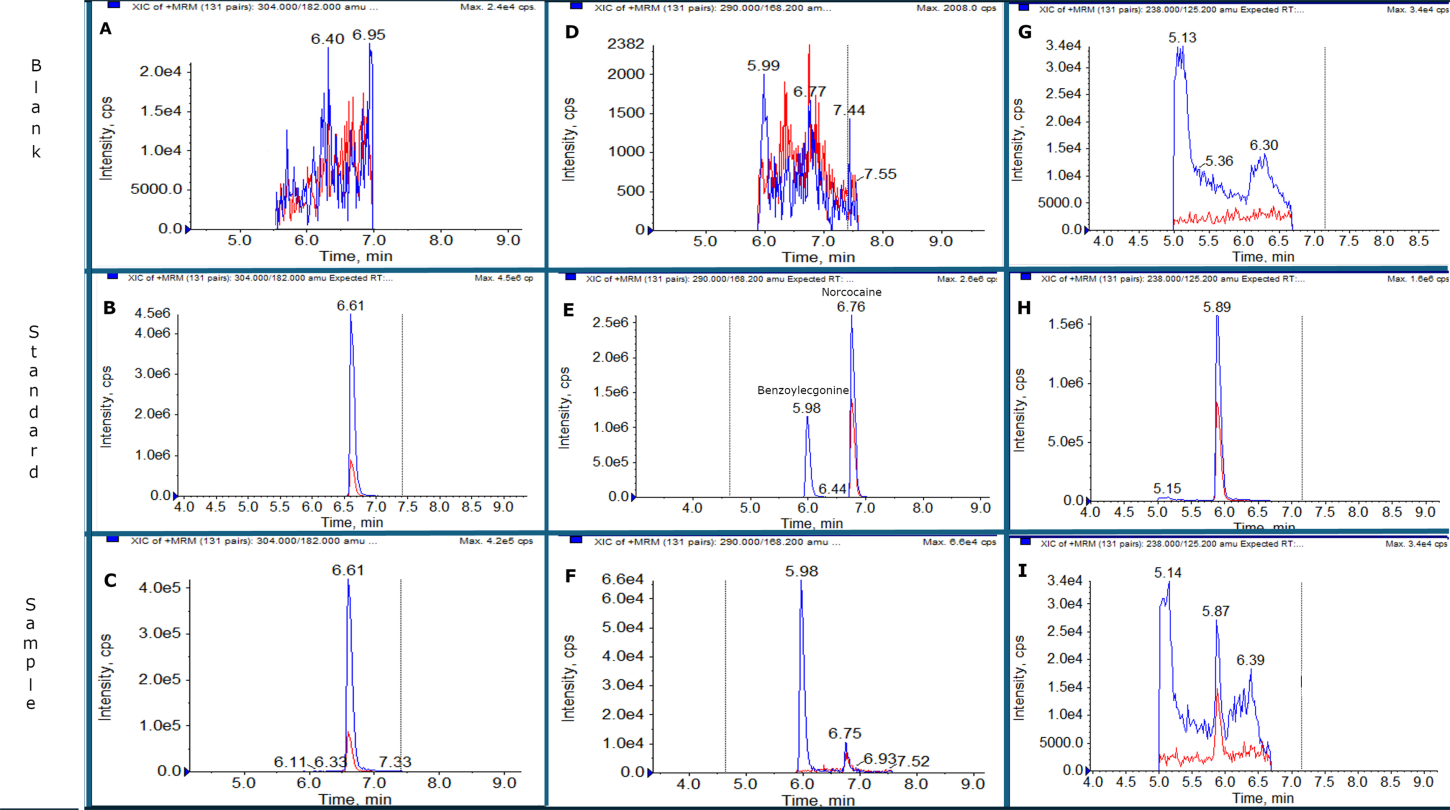
Chromatograms obtained from the analysis of real samples. A blank sample, a reference standard, and a real sample are shown for (A–C) cocaine, (D–F) benzoylecgonine and norcocaine, and (G–I) ketamine.

The procedure was tested on real samples arising from roadside drug testing campaigns for confirmatory analysis on positive screening. Out of a total of four samples tested, three tested positive for multiple drugs. Cocaine and its metabolites were present in all samples, while ketamine was detected in two of them; examples are shown in Figure 5.

The results show the applicability of the methodology on real samples.

## 4 Conclusions

In this paper, we have presented for the first time a LC–MS/MS method for the simultaneous identification and quantification of several classic drugs of abuse and NPS using a new liquid‐phase microextraction method (PALME) for extraction and clean‐up of OF samples. Due to the different chemical–physical characteristics of the examined substances, an experimental design was used to optimize the parameters for their determination, by employing a comprehensive full factorial design that encompassed three essential factors: pH of the donor solution, extraction solvent, and acceptor solution composition.

The results suggest that the developed method is suitable for the determination of 50 illicit drugs, belonging to different chemical classes and exhibited a good analytical performance allowing the use of OF as suitable matrix for drug testing. The method was also validated according to SWGTOX guidelines, and the measured parameters were generally found within the imposed limits; LOQ ranged from 0.03 to 5 ng mL^−1^.

A microextraction technique, like PALME, provides different advantages such as : the consumption of organic solvent is reduced to a few microliters per sample; the final solution (acceptor) is LC–MS/MS compatible; a high enrichment factor is obtained, with low ME; a low price linked to the possibility of processing up to 96 different samples at the same time.

The plates are commercially available, and the technique can be automated in a high throughput mode. A high enrichment can be obtained and compared with PP that is commonly used for clean‐up; PALME generally offers a better sample clean‐up avoiding ion suppression and reducing contamination. Applications involved pharmaceutical industry, hospital forensic toxicology, and doping laboratories; this technique could also be implemented in routine laboratories in the next future.

## Conflicts of Interest

The authors declare no conflicts of interest.

## Supporting information


**Data S1** Supplementary Information.

**Table S1** Full factorial response data analyzed in the laboratory. The correspondences among numbers and analytes are (1) 25I–NBOMe, (2) 2C‐B, (3) 2C‐H, (4) 2C‐T‐4, (5) 2C‐T‐7, (6) 2‐FMC, (7) 2‐MeOMet‐cathinone, (8) 4‐MethEt‐cathinone, (9) 6‐MAM, (10) AB005, (11) acetylfentanyl, (12) acrylfentanyl, (13) alfentanyl, (14) α‐methyl‐fentanyl, (15) α‐methyl‐thiofentanyl, (16) α‐PVP, (17) alprazolam, (18) amphetamine, (19) benzoylecgonine, (20) β‐hydroxy‐fentanyl, (21) buphedrone, (22) buprenorphine, (23) butylone, (24) butyrylfentanyl, (25) (±)*cis*‐3‐methyl‐fentanyl, (26) (±)*cis*‐3‐methyl‐thiofentanyl, (27) clonazepam, (28) codeine, (29) cocaine, (30) diethylproprion, (31) fentanyl, (32) fluorofentanyl, (33) furanylfentanyl, (34) JWH200, (35) ketamine, (36) lormetazepam, (37) MDA, (38) MDEA, (39) MDMA, (40) MDPV, (41) mephedrone, (42) methadone, (43) methamphetamine, (44) methylone, (45) methoxetamine, (46) morphine, (47) norbuprenorphine, (48) norcocaine, (49) norfentanyl, (50) ocfentanyl, (51) oxazepam, (52) phencyclidine, (53) remifentanyl, (54) sufentanyl, (55) triazolam, and (56) WIN‐55,212.

## Data Availability

The data that support the findings of this study are available from the corresponding author upon reasonable request.
