# Supplementary material for: Optimization of Parallel Artificial Liquid Membrane Extraction for the Determination of Over 50 Psychoactive Substances in Oral Fluid Through UHPLC–MS/MS
Source: Drug Test Anal. 2025 Apr 10;17(10):1877–95. doi: 10.1002/dta.3894 (PMC12489298; doi:10.1002/dta.3894)
Supplement: Supplementary file 1 — Data S1 Supplementary Information. [file DTA-17-1877-s001.docx]

---

output: html_document

editor_options:

chunk_output_type: console

---

#libraries

```{r}

library(rsm)

library(graphics)

library(desirability)

```

#import dataset

```{r}

library(dplyr)

XY<- read.csv("Xmean56New.csv", header= TRUE, sep=",")

str(XY)

X <- data.frame(XY[ , 2:57],row.names = XY$cond)

Xn<- X

# Identify numerical columns

numerical_cols <- sapply(Xn, is.numeric)

# Find the overall maximum value across all numerical columns

overall_max <- max(Xn[, numerical_cols], na.rm = TRUE)

# Normalize numerical columns using the overall maximum

Xn[, numerical_cols] <- Xn[, numerical_cols] / overall_max

str(Xn)

summary(Xn)

```

# Scaled full factorial design with responses

```{r}

# Define the original factor levels (unflag for using the level needed)

#original_levels <- factor(c(9, 10, 11, 12))

original_levels <- factor(c(0, 10, 20, 30))

# Define the corresponding new levels between -1 and 1

new_levels <- seq(-1, 1, length.out = length(levels(original_levels)))

# Perform the transformation

transformed_data <- as.factor(new_levels[as.integer(original_levels)])

# Levels of the transformed factor

levels(transformed_data)

#full 3 factors

factors3F <- expand.grid(x1 = c(-1, -0.333, 0.333, 1),

solv = c("DoA", "DiE", "DeC"),

x3 = c(-1, -0.333, 0.333, 1))

responses_All<- Xn

data_combined <- cbind(factors3F, responses_All)

# Split the data based on logP_solvent levels

split_datasets <- split(data_combined, data_combined$solv)

# IMPORTANT: change in DiE or DiE or DiE for the other solvent levels

dataOne<-split_datasets[["DiE"]]

factors<-dataOne[,c(1,3)]

responses<-dataOne[,c(4:59)]

str(factors)

str(responses)

colnames(responses)<-c("Y1","Y2","Y3","Y4","Y5","Y6","Y7","Y8","Y9","Y10","Y11","Y12","Y13","Y14","Y15","Y16","Y17","Y18","Y19","Y20","Y21","Y22","Y23","Y24","Y25","Y26","Y27","Y28","Y29","Y30","Y31","Y32","Y33","Y34","Y35","Y36","Y37","Y38","Y39","Y40","Y41","Y42","Y43","Y44","Y45","Y46","Y47","Y48","Y49","Y50","Y51","Y52","Y53","Y54","Y55","Y56")

data_palme<-cbind(factors,responses)

```

# 3D predict meth-pH

```{r}

# Create a meshgrid of pH and methanol levels

num_responses <- 56

factors_All <- expand.grid(pH = c(9, 10, 11, 12),

solv = c(1, 2, 3),#("DoA", "DiE", "DeC")

methanol = c(0, 10, 20, 30))

pH_vals <- seq(9, 12, length.out = 50)

methanol_vals <- seq(0, 30, length.out = 50)

grid1 <- expand.grid(pH = pH_vals, solv = 1, methanol = methanol_vals)

grid2 <- expand.grid(pH = pH_vals, solv = 2, methanol = methanol_vals)

grid3 <- expand.grid(pH = pH_vals, solv = 3, methanol = methanol_vals)

# Calculate predicted responses for the grid at solv DoA

predicted_responses_solv1 <- matrix(0, nrow = length(pH_vals), ncol = length(methanol_vals))

for (i in 1:num_responses) {

rsm_model <- rsm(responses_All[, i] ~ SO(pH, solv, methanol), data = factors_All)

predicted_response <- predict(rsm_model, newdata = grid1)

predicted_responses_solv1 <- predicted_responses_solv1 + matrix(predicted_response, nrow = length(pH_vals), ncol = length(methanol_vals))

}

# Calculate predicted responses for the grid at solv DiE

predicted_responses_solv2 <- matrix(0, nrow = length(pH_vals), ncol = length(methanol_vals))

for (i in 1:num_responses) {

rsm_model <- rsm(responses_All[, i] ~ SO(pH, solv, methanol), data = factors_All)

predicted_response <- predict(rsm_model, newdata = grid2)

predicted_responses_solv2 <- predicted_responses_solv2 + matrix(predicted_response, nrow = length(pH_vals), ncol = length(methanol_vals))

}

# Calculate predicted responses for the grid at solv DeC

predicted_responses_solv3 <- matrix(0, nrow = length(pH_vals), ncol = length(methanol_vals))

for (i in 1:num_responses) {

rsm_model <- rsm(responses_All[, i] ~ SO(pH, solv, methanol), data = factors_All)

predicted_response <- predict(rsm_model, newdata = grid3)

predicted_responses_solv3 <- predicted_responses_solv3 + matrix(predicted_response, nrow = length(pH_vals), ncol = length(methanol_vals))

}

# Create the 3D plots using persp

par (mfrow=c(1,3))

fig1<-persp(pH_vals, methanol_vals, predicted_responses_solv1, theta = 30, phi = 15,

col = "lightblue", border = "gray", shade = 0.5,

xlab = "pH", ylab = "methanol", zlab = "Responses DoA")

fig2<-persp(pH_vals, methanol_vals, predicted_responses_solv2, theta = 30, phi = 15,

col = "lightblue", border = "gray", shade = 0.5,

xlab = "pH", ylab = "methanol", zlab = "Responses DiE")

fig3<-persp(pH_vals, methanol_vals, predicted_responses_solv3, theta = 30, phi = 15,

col = "lightblue", border = "gray", shade = 0.5,

xlab = "pH", ylab = "methanol", zlab = "Responses DeC")

```

#rsm models for desirability optimization

```{r}

y1_m <- rsm(Y1 ~ SO(x1, x3), data = data_palme)

y2_m <- rsm(Y2 ~ SO(x1, x3), data = data_palme)

y3_m <- rsm(Y3 ~ SO(x1, x3), data = data_palme)

y4_m <- rsm(Y4 ~ SO(x1, x3), data = data_palme)

y5_m <- rsm(Y5 ~ SO(x1, x3), data = data_palme)

y6_m <- rsm(Y6 ~ SO(x1, x3), data = data_palme)

y7_m <- rsm(Y7 ~ SO(x1, x3), data = data_palme)

y8_m <- rsm(Y8 ~ SO(x1, x3), data = data_palme)

y9_m <- rsm(Y9 ~ SO(x1, x3), data = data_palme)

y10_m <- rsm(Y10 ~ SO(x1, x3), data = data_palme)

y11_m <- rsm(Y11 ~ SO(x1, x3), data = data_palme)

y12_m <- rsm(Y12 ~ SO(x1, x3), data = data_palme)

y13_m <- rsm(Y13 ~ SO(x1, x3), data = data_palme)

y14_m <- rsm(Y14 ~ SO(x1, x3), data = data_palme)

y15_m <- rsm(Y15 ~ SO(x1, x3), data = data_palme)

y16_m <- rsm(Y16 ~ SO(x1, x3), data = data_palme)

y17_m <- rsm(Y17 ~ SO(x1, x3), data = data_palme)

y18_m <- rsm(Y18 ~ SO(x1, x3), data = data_palme)

y19_m <- rsm(Y19 ~ SO(x1, x3), data = data_palme)

y20_m <- rsm(Y20 ~ SO(x1, x3), data = data_palme)

y21_m <- rsm(Y21 ~ SO(x1, x3), data = data_palme)

y22_m <- rsm(Y22 ~ SO(x1, x3), data = data_palme)

y23_m <- rsm(Y23 ~ SO(x1, x3), data = data_palme)

y24_m <- rsm(Y24 ~ SO(x1, x3), data = data_palme)

y25_m <- rsm(Y25 ~ SO(x1, x3), data = data_palme)

y26_m <- rsm(Y26 ~ SO(x1, x3), data = data_palme)

y27_m <- rsm(Y27 ~ SO(x1, x3), data = data_palme)

y28_m <- rsm(Y28 ~ SO(x1, x3), data = data_palme)

y29_m <- rsm(Y29 ~ SO(x1, x3), data = data_palme)

y30_m <- rsm(Y30 ~ SO(x1, x3), data = data_palme)

y31_m <- rsm(Y31 ~ SO(x1, x3), data = data_palme)

y32_m <- rsm(Y32 ~ SO(x1, x3), data = data_palme)

y33_m <- rsm(Y33 ~ SO(x1, x3), data = data_palme)

y34_m <- rsm(Y34 ~ SO(x1, x3), data = data_palme)

y35_m <- rsm(Y35 ~ SO(x1, x3), data = data_palme)

y36_m <- rsm(Y36 ~ SO(x1, x3), data = data_palme)

y37_m <- rsm(Y37 ~ SO(x1, x3), data = data_palme)

y38_m <- rsm(Y38 ~ SO(x1, x3), data = data_palme)

y39_m <- rsm(Y39 ~ SO(x1, x3), data = data_palme)

y40_m <- rsm(Y40 ~ SO(x1, x3), data = data_palme)

y41_m <- rsm(Y41 ~ SO(x1, x3), data = data_palme)

y42_m <- rsm(Y42 ~ SO(x1, x3), data = data_palme)

y43_m <- rsm(Y43 ~ SO(x1, x3), data = data_palme)

y44_m <- rsm(Y44 ~ SO(x1, x3), data = data_palme)

y45_m <- rsm(Y45 ~ SO(x1, x3), data = data_palme)

y46_m <- rsm(Y46 ~ SO(x1, x3), data = data_palme)

y47_m <- rsm(Y47 ~ SO(x1, x3), data = data_palme)

y48_m <- rsm(Y48 ~ SO(x1, x3), data = data_palme)

y49_m <- rsm(Y49 ~ SO(x1, x3), data = data_palme)

y50_m <- rsm(Y50 ~ SO(x1, x3), data = data_palme)

y51_m <- rsm(Y51 ~ SO(x1, x3), data = data_palme)

y52_m <- rsm(Y52 ~ SO(x1, x3), data = data_palme)

y53_m <- rsm(Y53 ~ SO(x1, x3), data = data_palme)

y54_m <- rsm(Y54 ~ SO(x1, x3), data = data_palme)

y55_m <- rsm(Y55 ~ SO(x1, x3), data = data_palme)

y56_m <- rsm(Y56 ~ SO(x1, x3), data = data_palme)

```

#Defining functions for simultaneous optimization

```{r}

# Prediction function

rsm_opt <- function(x, dObject, space = "square"){

df <- data.frame(x1 = x[1], x3 = x[2])

y1<- predict(y1_m, df)

y2<- predict(y2_m, df)

y3<- predict(y3_m, df)

y4<- predict(y4_m, df)

y5<- predict(y5_m, df)

y6<- predict(y6_m, df)

y7<- predict(y7_m, df)

y8<- predict(y8_m, df)

y9<- predict(y9_m, df)

y10<- predict(y10_m, df)

y11<- predict(y11_m, df)

y12<- predict(y12_m, df)

y13<- predict(y13_m, df)

y14<- predict(y14_m, df)

y15<- predict(y15_m, df)

y16<- predict(y16_m, df)

y17<- predict(y17_m, df)

y18<- predict(y18_m, df)

y19<- predict(y19_m, df)

y20<- predict(y20_m, df)

y21<- predict(y21_m, df)

y22<- predict(y22_m, df)

y23<- predict(y23_m, df)

y24<- predict(y24_m, df)

y25<- predict(y25_m, df)

y26<- predict(y26_m, df)

y27<- predict(y27_m, df)

y28<- predict(y28_m, df)

y29<- predict(y29_m, df)

y30<- predict(y30_m, df)

y31<- predict(y31_m, df)

y32<- predict(y32_m, df)

y33<- predict(y33_m, df)

y34<- predict(y34_m, df)

y35<- predict(y35_m, df)

y36<- predict(y36_m, df)

y37<- predict(y37_m, df)

y38<- predict(y38_m, df)

y39<- predict(y39_m, df)

y40<- predict(y40_m, df)

y41<- predict(y41_m, df)

y42<- predict(y42_m, df)

y43<- predict(y43_m, df)

y44<- predict(y44_m, df)

y45<- predict(y45_m, df)

y46<- predict(y46_m, df)

y47<- predict(y47_m, df)

y48<- predict(y48_m, df)

y49<- predict(y49_m, df)

y50<- predict(y50_m, df)

y51<- predict(y51_m, df)

y52<- predict(y52_m, df)

y53<- predict(y53_m, df)

y54<- predict(y54_m, df)

y55<- predict(y55_m, df)

y56<- predict(y56_m, df)

out <- predict(dObject, data.frame(y1= y1, y2= y2, y3= y3, y4= y4, y5= y5, y6= y6, y7= y7, y8= y8, y9= y9, y10= y10, y11= y11, y12= y12, y13= y13, y14= y14, y15= y15, y16= y16, y17= y17, y18= y18, y19= y19, y20= y20, y21= y21, y22= y22, y23= y23, y24= y24, y25= y25, y26= y26, y27= y27, y28= y28, y29= y29, y30= y30, y31= y31, y32= y32, y33= y33, y34= y34, y35= y35, y36= y36, y37= y37, y38= y38, y39= y39, y40= y40, y41= y41, y42= y42, y43= y43, y44= y44, y45= y45, y46= y46, y47= y47, y48= y48, y49= y49, y50= y50, y51= y51, y52= y52, y53= y53, y54= y54, y55= y55, y56= y56))

if(space == "circular" & sqrt(sum(x^2)) > 1) out <- 0

else if(space == "square" & any(abs(x) > 1)) out <- 0

out

}

# Optimization function

maximize_overall <- function(int_1 = c(-1, 1),

int_3 = c(-1, 1),

dObject = NULL,

space = "square"){

searchGrid <- expand.grid(

x1 = seq(int_1[1], int_1[2], length.out = 5),

x3 = seq(int_3[1], int_3[2], length.out = 5)

)

for(i in 1:dim(searchGrid)[1]){

tmp <- optim(as.vector(searchGrid[i,]),

rsm_opt,

dObject = dObject,

space = space,

control = list(fnscale = -1))

if(i == 1) best <- tmp

if(tmp$value > best$value) best <- tmp

}

best

}

```

#Defining desirability

```{r}

# Define the target desirability values for 52 responses

D_y1 <- dTarget(0, 0.001, 1)

D_y2 <- dTarget(0, 0.001, 1)

D_y3 <- dTarget(0, 0.001, 1)

D_y4 <- dTarget(0, 0.001, 1)

D_y5 <- dTarget(0, 0.001, 1)

D_y6 <- dTarget(0, 0.001, 1)

D_y7 <- dTarget(0, 0.001, 1)

D_y8 <- dTarget(0, 0.001, 1)

D_y9 <- dTarget(0, 0.001, 1)

D_y10 <- dTarget(0, 0.001, 1)

D_y11 <- dTarget(0, 0.001, 1)

D_y12 <- dTarget(0, 0.001, 1)

D_y13 <- dTarget(0, 0.001, 1)

D_y14 <- dTarget(0, 0.001, 1)

D_y15 <- dTarget(0, 0.001, 1)

D_y16 <- dTarget(0, 0.001, 1)

D_y17 <- dTarget(0, 0.001, 1)

D_y18 <- dTarget(0, 0.001, 1)

D_y19 <- dTarget(0, 0.001, 1)

D_y20 <- dTarget(0, 0.001, 1)

D_y21 <- dTarget(0, 0.001, 1)

D_y22 <- dTarget(0, 0.001, 1)

D_y23 <- dTarget(0, 0.001, 1)

D_y24 <- dTarget(0, 0.001, 1)

D_y25 <- dTarget(0, 0.001, 1)

D_y26 <- dTarget(0, 0.001, 1)

D_y27 <- dTarget(0, 0.001, 1)

D_y28 <- dTarget(0, 0.001, 1)

D_y29 <- dTarget(0, 0.001, 1)

D_y30 <- dTarget(0, 0.001, 1)

D_y31 <- dTarget(0, 0.001, 1)

D_y32 <- dTarget(0, 0.001, 1)

D_y33 <- dTarget(0, 0.001, 1)

D_y34 <- dTarget(0, 0.001, 1)

D_y35 <- dTarget(0, 0.001, 1)

D_y36 <- dTarget(0, 0.001, 1)

D_y37 <- dTarget(0, 0.001, 1)

D_y38 <- dTarget(0, 0.001, 1)

D_y39 <- dTarget(0, 0.001, 1)

D_y40 <- dTarget(0, 0.001, 1)

D_y41 <- dTarget(0, 0.001, 1)

D_y42 <- dTarget(0, 0.001, 1)

D_y43 <- dTarget(0, 0.001, 1)

D_y44 <- dTarget(0, 0.001, 1)

D_y45 <- dTarget(0, 0.001, 1)

D_y46 <- dTarget(0, 0.001, 1)

D_y47 <- dTarget(0, 0.001, 1)

D_y48 <- dTarget(0, 0.001, 1)

D_y49 <- dTarget(0, 0.001, 1)

D_y50 <- dTarget(0, 0.001, 1)

D_y51 <- dTarget(0, 0.001, 1)

D_y52 <- dTarget(0, 0.001, 1)

D_y53 <- dTarget(0, 0.001, 1)

D_y54 <- dTarget(0, 0.001, 1)

D_y55 <- dTarget(0, 0.001, 1)

D_y56 <- dTarget(0, 0.001, 1)

#Global desirability is in turn defined by the previously defined functions.

D_overall <- dOverall(D_y1, D_y2, D_y3, D_y4, D_y5, D_y6, D_y7, D_y8, D_y9, D_y10, D_y11, D_y12, D_y13, D_y14, D_y15, D_y16, D_y17, D_y18, D_y19, D_y20, D_y21, D_y22, D_y23, D_y24, D_y25, D_y26, D_y27, D_y28, D_y29, D_y30, D_y31, D_y32, D_y33, D_y34, D_y35, D_y36, D_y37, D_y38, D_y39, D_y40, D_y41, D_y42, D_y43, D_y44, D_y45, D_y46, D_y47, D_y48, D_y49, D_y50, D_y51, D_y52, D_y53, D_y54, D_y55, D_y56)

```

#Carrying out simultaneous optimization

```{r}

overall_opt <- maximize_overall(dObject = D_overall)

```

#Obtaining predictions of each response in the global optimum

```{r}

# Optimal point as a data frame

data_opt <- data.frame(

x1 = overall_opt$par[1],

x3 = overall_opt$par[2]

)

# Predict each response at overall optimization point

y1_opt <- predict(y1_m, data_opt)

y2_opt <- predict(y2_m, data_opt)

y3_opt <- predict(y3_m, data_opt)

y4_opt <- predict(y4_m, data_opt)

y5_opt <- predict(y5_m, data_opt)

y6_opt <- predict(y6_m, data_opt)

y7_opt <- predict(y7_m, data_opt)

y8_opt <- predict(y8_m, data_opt)

y9_opt <- predict(y9_m, data_opt)

y10_opt <- predict(y10_m, data_opt)

y11_opt <- predict(y11_m, data_opt)

y12_opt <- predict(y12_m, data_opt)

y13_opt <- predict(y13_m, data_opt)

y14_opt <- predict(y14_m, data_opt)

y15_opt <- predict(y15_m, data_opt)

y16_opt <- predict(y16_m, data_opt)

y17_opt <- predict(y17_m, data_opt)

y18_opt <- predict(y18_m, data_opt)

y19_opt <- predict(y19_m, data_opt)

y20_opt <- predict(y20_m, data_opt)

y21_opt <- predict(y21_m, data_opt)

y22_opt <- predict(y22_m, data_opt)

y23_opt <- predict(y23_m, data_opt)

y24_opt <- predict(y24_m, data_opt)

y25_opt <- predict(y25_m, data_opt)

y26_opt <- predict(y26_m, data_opt)

y27_opt <- predict(y27_m, data_opt)

y28_opt <- predict(y28_m, data_opt)

y29_opt <- predict(y29_m, data_opt)

y30_opt <- predict(y30_m, data_opt)

y31_opt <- predict(y31_m, data_opt)

y32_opt <- predict(y32_m, data_opt)

y33_opt <- predict(y33_m, data_opt)

y34_opt <- predict(y34_m, data_opt)

y35_opt <- predict(y35_m, data_opt)

y36_opt <- predict(y36_m, data_opt)

y37_opt <- predict(y37_m, data_opt)

y38_opt <- predict(y38_m, data_opt)

y39_opt <- predict(y39_m, data_opt)

y40_opt <- predict(y40_m, data_opt)

y41_opt <- predict(y41_m, data_opt)

y42_opt <- predict(y42_m, data_opt)

y43_opt <- predict(y43_m, data_opt)

y44_opt <- predict(y44_m, data_opt)

y45_opt <- predict(y45_m, data_opt)

y46_opt <- predict(y46_m, data_opt)

y47_opt <- predict(y47_m, data_opt)

y48_opt <- predict(y48_m, data_opt)

y49_opt <- predict(y49_m, data_opt)

y50_opt <- predict(y50_m, data_opt)

y51_opt <- predict(y51_m, data_opt)

y52_opt <- predict(y52_m, data_opt)

y53_opt <- predict(y53_m, data_opt)

y54_opt <- predict(y54_m, data_opt)

y55_opt <- predict(y55_m, data_opt)

y56_opt <- predict(y56_m, data_opt)

# Response predictions in a data frame

res_opt <- data.frame(y1_opt, y2_opt, y3_opt, y4_opt, y5_opt, y6_opt, y7_opt, y8_opt, y9_opt, y10_opt, y11_opt, y12_opt, y13_opt, y14_opt, y15_opt, y16_opt, y17_opt, y18_opt, y19_opt, y20_opt, y21_opt, y22_opt, y23_opt, y24_opt, y25_opt, y26_opt, y27_opt, y28_opt, y29_opt, y30_opt, y31_opt, y32_opt, y33_opt, y34_opt, y35_opt, y36_opt, y37_opt, y38_opt, y39_opt, y40_opt, y41_opt, y42_opt, y43_opt, y44_opt, y45_opt, y46_opt, y47_opt, y48_opt, y49_opt, y50_opt, y51_opt, y52_opt, y53_opt, y54_opt, y55_opt, y56_opt)

rownames(res_opt) <- "Optimal responses"

round(res_opt, 5)

write.csv(res_opt, file = "res_optDiE56new.csv")

```

#Visualization of the desirability prediction

```{r}

# Generate a matrix with desirability predictions within the experimental region

par (mfrow=c(1,1))

d_matrix <- function(model_1, model_2, model_3, model_4, model_5, model_6, model_7, model_8, model_9, model_10, model_11, model_12, model_13, model_14, model_15, model_16, model_17, model_18, model_19, model_20, model_21, model_22, model_23, model_24, model_25, model_26, model_27, model_28, model_29, model_30, model_31, model_32, model_33, model_34, model_35, model_36, model_37, model_38, model_39, model_40, model_41, model_42, model_43, model_44, model_45, model_46, model_47, model_48, model_49, model_50, model_51, model_52, model_53, model_54, model_55, model_56,

dObject, l_x = c(-1, 1), by = 0.1){

x <- seq(l_x[1], l_x[2], by = by)

lx <- length(x)

data_x <- expand.grid(x1 = x, x3 = x)

y_1 <- predict(y1_m, data_x)

y_2 <- predict(y2_m, data_x)

y_3 <- predict(y3_m, data_x)

y_4 <- predict(y4_m, data_x)

y_5 <- predict(y5_m, data_x)

y_6 <- predict(y6_m, data_x)

y_7 <- predict(y7_m, data_x)

y_8 <- predict(y8_m, data_x)

y_9 <- predict(y9_m, data_x)

y_10 <- predict(y10_m, data_x)

y_11 <- predict(y11_m, data_x)

y_12 <- predict(y12_m, data_x)

y_13 <- predict(y13_m, data_x)

y_14 <- predict(y14_m, data_x)

y_15 <- predict(y15_m, data_x)

y_16 <- predict(y16_m, data_x)

y_17 <- predict(y17_m, data_x)

y_18 <- predict(y18_m, data_x)

y_19 <- predict(y19_m, data_x)

y_20 <- predict(y20_m, data_x)

y_21 <- predict(y21_m, data_x)

y_22 <- predict(y22_m, data_x)

y_23 <- predict(y23_m, data_x)

y_24 <- predict(y24_m, data_x)

y_25 <- predict(y25_m, data_x)

y_26 <- predict(y26_m, data_x)

y_27 <- predict(y27_m, data_x)

y_28 <- predict(y28_m, data_x)

y_29 <- predict(y29_m, data_x)

y_30 <- predict(y30_m, data_x)

y_31 <- predict(y31_m, data_x)

y_32 <- predict(y32_m, data_x)

y_33 <- predict(y33_m, data_x)

y_34 <- predict(y34_m, data_x)

y_35 <- predict(y35_m, data_x)

y_36 <- predict(y36_m, data_x)

y_37 <- predict(y37_m, data_x)

y_38 <- predict(y38_m, data_x)

y_39 <- predict(y39_m, data_x)

y_40 <- predict(y40_m, data_x)

y_41 <- predict(y41_m, data_x)

y_42 <- predict(y42_m, data_x)

y_43 <- predict(y43_m, data_x)

y_44 <- predict(y44_m, data_x)

y_45 <- predict(y45_m, data_x)

y_46 <- predict(y46_m, data_x)

y_47 <- predict(y47_m, data_x)

y_48 <- predict(y48_m, data_x)

y_49 <- predict(y49_m, data_x)

y_50 <- predict(y50_m, data_x)

y_51 <- predict(y51_m, data_x)

y_52 <- predict(y52_m, data_x)

y_53 <- predict(y53_m, data_x)

y_54 <- predict(y54_m, data_x)

y_55 <- predict(y55_m, data_x)

y_56 <- predict(y56_m, data_x)

d_m <- predict(D_overall, data.frame(Y1 = y_1, Y2 = y_2, Y3 = y_3, Y4 = y_4, Y5 = y_5, Y6 = y_6, Y7 = y_7, Y8 = y_8, Y9 = y_9, Y10 = y_10, Y11 = y_11, Y12 = y_12, Y13 = y_13, Y14 = y_14, Y15 = y_15, Y16 = y_16, Y17 = y_17, Y18 = y_18, Y19 = y_19, Y20 = y_20, Y21 = y_21, Y22 = y_22, Y23 = y_23, Y24 = y_24, Y25 = y_25, Y26 = y_26, Y27 = y_27, Y28 = y_28, Y29 = y_29, Y30 = y_30, Y31 = y_31, Y32 = y_32, Y33 = y_33, Y34 = y_34, Y35 = y_35, Y36 = y_36, Y37 = y_37, Y38 = y_38, Y39 = y_39, Y40 = y_40, Y41 = y_41, Y42 = y_42, Y43 = y_43, Y44 = y_44, Y45 = y_45, Y46 = y_46, Y47 = y_47, Y48 = y_48, Y49 = y_49, Y50 = y_50, Y51 = y_51, Y52 = y_52, Y53 = y_53, Y54 = y_54, Y55 = y_55, Y56 = y_56))

dim(d_m) <- c(lx, lx)

list(d_m = d_m, x = x)

}

# Deploys a contour plot for desirability within the experimental region

contour_d <- function(data = NULL, main = " ", xlab = "x1", ylab = "x2"){

filled.contour(

z = data$d_m, x = data$x, y = data$x,

color.palette = colorRamps::matlab.like,

plot.title = title(main = main, xlab = xlab, ylab = ylab, cex.lab = 1,

cex.main = 1),

plot.axes = {

axis(1, cex.axis = 1)

axis(2, cex.axis = 1)

}

)

}

# Desirability plot

dpx3_1 <- d_matrix(y1_m, y2_m, y3_m, y4_m, D_overall,

l_x = c(-1, 1))

contour_d(dpx3_1, main = "x2 = Solv")

```

#Original pH and methanol values using reverse-scale

```{r}

# Define the known points for pH

scaled_pointspH <- c(-1, -0.33, 0.33, 1)

original_pointspH <- c(9, 10, 11, 12)

scaled_valuepH <- data_opt[1,1]

original_pH <- approx(scaled_pointspH, original_pointspH, xout = scaled_valuepH)$y

# Define the known points for methanol

scaled_pointsMet <- c(-1, -0.33, 0.33, 1)

original_pointsMet <- c(0, 10, 20, 30)

scaled_valueMet <- data_opt[1,2]

original_met <- approx(scaled_pointsMet, original_pointsMet, xout = scaled_valueMet)$y

```

#print results

```{r}

print("overall optimization info")

overall_opt$par

overall_opt$value

overall_opt$counts

overall_opt$convergence

overall_opt$message

print ("data_opt")

data_opt

print ("the original pH value")

original_pH

original_met

print ("Optimal responses")

rownames(res_opt) <- "Optimal responses"

round(res_opt, 5)

```

#capture output

```{r}

capture.output(

print("overall info"),

overall_opt$par,

overall_opt$value,

overall_opt$counts,

overall_opt$convergence,

overall_opt$message,

print ("data_opt"),

data_opt,

print ("the original pH and met value"),

original_pH,

original_met,

print ("Optimal responses"),

round(res_opt, 5),

file = "Areas2F_DiE56new.txt")

```
