# Supplementary material for: Optimization of Parallel Artificial Liquid Membrane Extraction for the Determination of Over 50 Psychoactive Substances in Oral Fluid Through UHPLC–MS/MS
Source: Drug Test Anal. 2025 Apr 10;17(10):1877–95. doi: 10.1002/dta.3894 (PMC12489298; doi:10.1002/dta.3894)
Supplement: Supplementary file 2 — Table S1 Full factorial response data analyzed in the laboratory. The correspondences among numbers and analytes are (1) 25I–NBOMe, (2) 2C‐B, (3) 2C‐H, (4) 2C‐T‐4, (5) 2C‐T‐7, (6) 2‐FMC, (7) 2‐MeOMet‐cathinone, (8) 4‐MethEt‐cathinone, (9) 6‐MAM, (10) AB005, (11) acetylfentanyl, (12) acrylfentanyl, (13) alfentanyl, (14) α‐methyl‐fentanyl, (15) α‐methyl‐thiofentanyl, (16) α‐PVP, (17) alprazolam, (18) amphetamine, (19) benzoylecgonine, (20) β‐hydroxy‐fentanyl, (21) buphedrone, (22) buprenorphine, (23) butylone, (24) butyrylfentanyl, (25) (±)cis‐3‐methyl‐fentanyl, (26) (±)cis‐3‐methyl‐thiofentanyl, (27) clonazepam, (28) codeine, (29) cocaine, (30) diethylproprion, (31) fentanyl, (32) fluorofentanyl, (33) furanylfentanyl, (34) JWH200, (35) ketamine, (36) lormetazepam, (37) MDA, (38) MDEA, (39) MDMA, (40) MDPV, (41) mephedrone, (42) methadone, (43) methamphetamine, (44) methylone, (45) methoxetamine, (46) morphine, (47) norbuprenorphine, (48) norcocaine, (49) norfentanyl, (50) ocfentanyl, (51) oxazepam, (52) phencyclidine, (53) remifentanyl, (54) sufentanyl, (55) triazolam, and (56) WIN‐55,212. [file DTA-17-1877-s002.docx]

| **pH** | **MeOH (%)** | **Solvent** | **Test** | Analytes* | | | | | | | | | | | | | | | | | | | | | | | | | | | | | | | | | | | | | | | | | | | | | | | | | | | | | | |  |
| --- | --- | --- | --- | --- | --- | --- | --- | --- | --- | --- | --- | --- | --- | --- | --- | --- | --- | --- | --- | --- | --- | --- | --- | --- | --- | --- | --- | --- | --- | --- | --- | --- | --- | --- | --- | --- | --- | --- | --- | --- | --- | --- | --- | --- | --- | --- | --- | --- | --- | --- | --- | --- | --- | --- | --- | --- | --- | --- | --- |
|  |  |  |  | 1 | 2 | 3 | 4 | 5 | 6 | 7 | 8 | 9 | 10 | 11 | 12 | 13 | 14 | 15 | 16 | 17 | 18 | 19 | 20 | 21 | 22 | 23 | 24 | 25 | 26 | 27 | 28 | 29 | 30 | 31 | 32 | 33 | 34 | 35 | 36 | 37 | 38 | 39 | 40 | 41 | 42 | 43 | 44 | 45 | 46 | 47 | 48 | 49 | 50 | 51 | 52 | 53 | 54 | 55 | 56 |
| 9 | 0 | DoA | A1 | 1,26E+07 | 0,00E+00 | 0,00E+00 | 0,00E+00 | 0,00E+00 | 8,64E+04 | 1,16E+04 | 1,80E+05 | 1,32E+04 | 7,14E+06 | 3,71E+06 | 1,60E+07 | 3,22E+06 | 1,15E+07 | 7,51E+06 | 6,50E+05 | 4,85E+06 | 0,00E+00 | 0,00E+00 | 1,09E+06 | 2,10E+05 | 7,14E+05 | 1,15E+05 | 5,93E+07 | 5,13E+07 | 1,88E+07 | 2,28E+06 | 0,00E+00 | 7,13E+05 | 2,30E+06 | 6,30E+07 | 3,53E+07 | 2,62E+07 | 5,98E+05 | 4,11E+06 | 3,77E+06 | 0,00E+00 | 0,00E+00 | 0,00E+00 | 2,75E+06 | 8,08E+04 | 2,30E+05 | 0,00E+00 | 2,14E+04 | 6,86E+05 | 0,00E+00 | 0,00E+00 | 5,65E+05 | 0,00E+00 | 1,16E+06 | 5,35E+05 | 1,46E+06 | 1,21E+05 | 1,55E+07 | 7,47E+06 | 9,85E+04 |
| 10 | 0 | DoA | A2 | 5,00E+06 | 4,76E+04 | 3,29E+04 | 6,07E+04 | 1,18E+05 | 3,73E+04 | 2,45E+04 | 8,73E+04 | 7,45E+03 | 2,09E+06 | 1,88E+06 | 5,85E+06 | 1,54E+06 | 4,16E+06 | 2,92E+06 | 1,11E+05 | 3,92E+06 | 0,00E+00 | 0,00E+00 | 4,43E+05 | 1,06E+05 | 3,68E+05 | 5,14E+04 | 3,05E+07 | 2,20E+06 | 9,19E+06 | 2,05E+06 | 0,00E+00 | 1,51E+05 | 2,44E+05 | 2,52E+07 | 1,42E+07 | 9,85E+06 | 2,70E+05 | 7,68E+05 | 2,53E+06 | 1,63E+04 | 2,40E+04 | 0,00E+00 | 2,96E+05 | 9,06E+04 | 2,62E+05 | 0,00E+00 | 1,64E+04 | 2,05E+05 | 0,00E+00 | 6,32E+03 | 2,26E+05 | 8,95E+03 | 4,48E+05 | 3,15E+05 | 2,37E+05 | 5,22E+04 | 7,67E+06 | 3,45E+06 | 7,10E+04 |
| 11 | 0 | DoA | A3 | 5,71E+06 | 6,98E+04 | 8,36E+04 | 8,86E+04 | 1,38E+05 | 6,13E+04 | 5,96E+04 | 7,81E+04 | 0,00E+00 | 2,52E+06 | 1,03E+06 | 3,67E+06 | 9,52E+05 | 2,87E+06 | 1,80E+06 | 5,95E+04 | 5,44E+06 | 3,91E+04 | 2,22E+04 | 2,60E+05 | 1,33E+05 | 2,60E+05 | 1,09E+05 | 1,81E+07 | 1,08E+06 | 6,01E+06 | 2,21E+06 | 0,00E+00 | 1,43E+05 | 1,38E+05 | 1,59E+07 | 9,49E+06 | 6,63E+06 | 3,85E+05 | 4,27E+05 | 2,90E+06 | 5,21E+04 | 7,74E+04 | 0,00E+00 | 1,93E+05 | 2,23E+05 | 2,17E+05 | 9,21E+04 | 4,90E+04 | 1,52E+05 | 0,00E+00 | 1,03E+04 | 1,72E+05 | 2,27E+04 | 2,43E+05 | 3,94E+05 | 1,89E+05 | 2,73E+04 | 5,49E+06 | 3,77E+06 | 1,12E+05 |
| 12 | 0 | DoA | A4 | 7,17E+06 | 1,57E+05 | 8,84E+04 | 1,80E+05 | 3,22E+05 | 7,69E+05 | 2,65E+05 | 2,11E+06 | 9,55E+04 | 2,30E+06 | 7,04E+06 | 9,56E+06 | 2,96E+06 | 6,20E+06 | 3,52E+06 | 8,15E+05 | 4,97E+06 | 7,51E+04 | 5,95E+03 | 1,81E+06 | 1,80E+06 | 3,04E+05 | 1,11E+06 | 3,61E+07 | 2,13E+06 | 7,85E+06 | 1,91E+06 | 6,73E+03 | 2,13E+06 | 3,73E+06 | 4,15E+07 | 1,75E+07 | 1,33E+07 | 4,20E+05 | 5,10E+06 | 2,79E+06 | 7,50E+04 | 1,80E+05 | 4,70E+04 | 3,25E+06 | 1,93E+06 | 4,62E+05 | 2,42E+05 | 2,92E+05 | 3,03E+06 | 6,31E+03 | 2,80E+04 | 3,47E+06 | 3,42E+04 | 2,88E+06 | 3,37E+05 | 1,10E+06 | 2,71E+05 | 7,61E+06 | 3,82E+06 | 1,07E+05 |
| 9 | 0 | DiE | A5 | 1,18E+07 | 0,00E+00 | 0,00E+00 | 0,00E+00 | 0,00E+00 | 6,65E+04 | 1,17E+04 | 1,16E+05 | 0,00E+00 | 6,48E+06 | 3,44E+06 | 1,44E+07 | 2,81E+06 | 1,10E+07 | 6,57E+06 | 1,67E+06 | 5,05E+06 | 5,78E+05 | 2,78E+05 | 9,86E+05 | 2,05E+05 | 6,71E+05 | 1,41E+05 | 7,35E+07 | 4,70E+07 | 1,83E+07 | 2,38E+06 | 0,00E+00 | 1,24E+07 | 2,38E+06 | 8,02E+07 | 3,86E+07 | 3,15E+07 | 7,84E+05 | 1,03E+07 | 3,01E+06 | 6,40E+05 | 0,00E+00 | 0,00E+00 | 2,66E+06 | 9,67E+04 | 1,89E+05 | 0,00E+00 | 1,69E+06 | 7,11E+05 | 0,00E+00 | 0,00E+00 | 6,05E+05 | 1,72E+04 | 1,07E+06 | 4,70E+05 | 3,60E+06 | 1,06E+05 | 1,56E+07 | 6,39E+06 | 6,56E+04 |
| 10 | 0 | DiE | A6 | 1,38E+07 | 0,00E+00 | 0,00E+00 | 0,00E+00 | 0,00E+00 | 8,74E+04 | 1,21E+04 | 9,19E+04 | 8,05E+03 | 7,33E+06 | 3,38E+06 | 1,60E+07 | 3,32E+06 | 1,17E+07 | 7,78E+06 | 6,57E+05 | 4,96E+06 | 0,00E+00 | 2,85E+03 | 9,96E+05 | 2,08E+05 | 7,81E+05 | 1,06E+05 | 6,97E+07 | 5,06E+07 | 1,81E+07 | 2,29E+06 | 0,00E+00 | 6,89E+05 | 2,23E+06 | 6,00E+07 | 3,45E+07 | 2,73E+07 | 6,30E+05 | 4,18E+06 | 3,90E+06 | 3,31E+03 | 0,00E+00 | 0,00E+00 | 2,61E+06 | 1,17E+05 | 2,30E+05 | 0,00E+00 | 1,70E+04 | 6,09E+05 | 0,00E+00 | 0,00E+00 | 5,46E+05 | 0,00E+00 | 1,17E+06 | 5,25E+05 | 1,52E+06 | 1,11E+05 | 1,68E+07 | 7,77E+06 | 9,94E+04 |
| 11 | 0 | DiE | A7 | 1,30E+07 | 4,14E+05 | 2,53E+05 | 3,98E+05 | 6,80E+05 | 5,20E+06 | 1,37E+06 | 7,85E+06 | 2,71E+05 | 6,06E+06 | 1,62E+07 | 2,08E+07 | 5,26E+06 | 1,51E+07 | 8,26E+06 | 1,94E+06 | 5,70E+06 | 2,07E+05 | 0,00E+00 | 4,13E+06 | 9,06E+06 | 6,53E+05 | 7,06E+06 | 8,40E+07 | 5,72E+07 | 1,91E+07 | 2,22E+06 | 0,00E+00 | 9,16E+06 | 8,13E+06 | 9,26E+07 | 3,95E+07 | 3,01E+07 | 5,92E+05 | 1,11E+07 | 3,36E+06 | 2,75E+05 | 1,47E+06 | 2,30E+05 | 9,57E+06 | 8,18E+06 | 1,54E+05 | 1,19E+06 | 1,79E+06 | 1,03E+07 | 0,00E+00 | 7,01E+04 | 1,06E+07 | 1,27E+04 | 7,09E+06 | 4,42E+05 | 2,71E+06 | 7,16E+05 | 1,63E+07 | 6,09E+06 | 9,28E+04 |
| 12 | 0 | DiE | A8 | 9,75E+06 | 0,00E+00 | 0,00E+00 | 0,00E+00 | 0,00E+00 | 3,38E+04 | 3,87E+03 | 4,70E+04 | 0,00E+00 | 7,28E+06 | 1,34E+06 | 9,20E+06 | 1,01E+06 | 7,14E+06 | 5,78E+06 | 4,53E+05 | 1,02E+06 | 0,00E+00 | 3,01E+03 | 2,94E+05 | 1,11E+05 | 6,93E+05 | 4,00E+04 | 5,93E+07 | 5,34E+07 | 1,76E+07 | 4,34E+06 | 0,00E+00 | 2,84E+05 | 1,50E+06 | 3,39E+07 | 2,66E+07 | 1,69E+07 | 1,40E+06 | 1,64E+06 | 9,45E+06 | 0,00E+00 | 0,00E+00 | 0,00E+00 | 1,33E+06 | 0,00E+00 | 4,28E+05 | 0,00E+00 | 0,00E+00 | 3,10E+05 | 1,44E+03 | 0,00E+00 | 1,89E+05 | 0,00E+00 | 3,27E+05 | 7,70E+05 | 1,34E+06 | 2,74E+04 | 1,52E+07 | 4,43E+06 | 8,99E+05 |
| 9 | 0 | DeC | A9 | 1,13E+07 | 0,00E+00 | 0,00E+00 | 0,00E+00 | 0,00E+00 | 6,09E+04 | 5,80E+03 | 8,79E+04 | 0,00E+00 | 8,00E+06 | 1,95E+06 | 1,22E+07 | 1,20E+06 | 9,28E+06 | 6,98E+06 | 7,07E+05 | 1,29E+06 | 0,00E+00 | 6,24E+03 | 4,32E+05 | 1,86E+05 | 7,64E+05 | 6,30E+04 | 7,04E+07 | 5,95E+07 | 1,94E+07 | 4,86E+06 | 0,00E+00 | 4,82E+05 | 2,36E+06 | 4,56E+07 | 3,20E+07 | 2,14E+07 | 1,52E+06 | 2,58E+06 | 9,23E+06 | 0,00E+00 | 0,00E+00 | 0,00E+00 | 2,14E+06 | 0,00E+00 | 8,18E+04 | 0,00E+00 | 9,56E+03 | 4,92E+05 | 0,00E+00 | 0,00E+00 | 3,02E+05 | 0,00E+00 | 4,79E+05 | 8,71E+05 | 1,90E+06 | 4,15E+04 | 1,66E+07 | 4,70E+06 | 9,88E+05 |
| 10 | 0 | DeC | A10 | 7,91E+06 | 0,00E+00 | 0,00E+00 | 0,00E+00 | 0,00E+00 | 2,75E+04 | 5,82E+03 | 3,20E+04 | 0,00E+00 | 6,99E+06 | 1,31E+06 | 9,23E+06 | 9,82E+05 | 7,05E+06 | 5,60E+06 | 4,81E+05 | 5,82E+05 | 0,00E+00 | 0,00E+00 | 3,23E+05 | 7,84E+04 | 6,61E+05 | 3,18E+04 | 4,12E+07 | 4,18E+07 | 1,66E+07 | 3,26E+06 | 0,00E+00 | 2,85E+05 | 1,54E+06 | 3,41E+07 | 2,53E+07 | 1,58E+07 | 1,34E+06 | 1,59E+06 | 5,63E+06 | 0,00E+00 | 0,00E+00 | 0,00E+00 | 1,37E+06 | 0,00E+00 | 4,82E+04 | 0,00E+00 | 0,00E+00 | 3,00E+05 | 0,00E+00 | 0,00E+00 | 1,71E+05 | 0,00E+00 | 3,16E+05 | 5,34E+05 | 1,12E+06 | 2,74E+04 | 1,42E+07 | 2,45E+06 | 8,46E+05 |
| 11 | 0 | DeC | A11 | 1,37E+07 | 1,43E+05 | 0,00E+00 | 1,99E+05 | 3,75E+05 | 2,94E+06 | 5,78E+05 | 6,88E+06 | 5,22E+04 | 6,87E+06 | 1,54E+07 | 1,99E+07 | 2,51E+06 | 1,44E+07 | 7,69E+06 | 1,84E+06 | 8,95E+05 | 1,15E+05 | 0,00E+00 | 3,67E+06 | 6,30E+06 | 7,02E+05 | 2,75E+06 | 7,88E+07 | 5,21E+07 | 1,75E+07 | 3,69E+06 | 0,00E+00 | 8,59E+06 | 6,90E+06 | 8,48E+07 | 3,64E+07 | 2,85E+07 | 1,37E+06 | 8,91E+06 | 5,55E+06 | 9,88E+04 | 9,58E+05 | 9,91E+04 | 9,13E+06 | 3,85E+06 | 3,12E+04 | 8,46E+05 | 6,21E+05 | 8,88E+06 | 0,00E+00 | 2,95E+04 | 7,93E+06 | 7,66E+03 | 6,25E+06 | 7,11E+05 | 2,68E+06 | 2,51E+05 | 1,55E+07 | 3,45E+06 | 9,04E+05 |
| 12 | 0 | DeC | A12 | 7,26E+06 | 8,25E+04 | 0,00E+00 | 9,92E+04 | 2,04E+05 | 5,61E+04 | 3,40E+04 | 1,62E+05 | 1,16E+04 | 4,76E+06 | 3,30E+06 | 9,12E+06 | 2,59E+06 | 6,48E+06 | 4,54E+06 | 1,97E+05 | 4,33E+06 | 1,75E+04 | 1,12E+04 | 7,48E+05 | 1,63E+05 | 5,41E+05 | 7,25E+04 | 4,61E+07 | 3,49E+07 | 1,34E+07 | 3,27E+06 | 4,20E+03 | 2,43E+05 | 4,57E+05 | 4,01E+07 | 2,07E+07 | 1,50E+07 | 5,36E+05 | 1,55E+06 | 2,94E+06 | 2,55E+04 | 4,14E+04 | 0,00E+00 | 5,91E+05 | 1,55E+05 | 1,36E+05 | 0,00E+00 | 2,82E+04 | 3,95E+05 | 5,05E+03 | 1,22E+04 | 4,16E+05 | 1,07E+04 | 7,86E+05 | 3,45E+05 | 3,93E+05 | 9,32E+04 | 1,04E+07 | 4,32E+06 | 1,50E+05 |
| 9 | 10 | DoA | B1 | 1,40E+07 | 0,00E+00 | 0,00E+00 | 0,00E+00 | 0,00E+00 | 8,59E+04 | 9,82E+03 | 1,25E+05 | 0,00E+00 | 7,62E+06 | 3,27E+06 | 1,61E+07 | 3,44E+06 | 1,20E+07 | 8,00E+06 | 6,45E+05 | 5,44E+06 | 0,00E+00 | 3,72E+03 | 9,98E+05 | 1,92E+05 | 8,06E+05 | 1,39E+05 | 8,09E+07 | 5,92E+07 | 1,99E+07 | 3,14E+06 | 0,00E+00 | 6,67E+05 | 2,78E+06 | 6,10E+07 | 3,66E+07 | 2,73E+07 | 6,53E+05 | 4,66E+06 | 4,80E+06 | 0,00E+00 | 0,00E+00 | 0,00E+00 | 2,51E+06 | 1,54E+05 | 2,22E+05 | 0,00E+00 | 1,52E+04 | 6,25E+05 | 0,00E+00 | 0,00E+00 | 5,07E+05 | 0,00E+00 | 1,10E+06 | 6,47E+05 | 1,49E+06 | 1,11E+05 | 1,61E+07 | 9,75E+06 | 1,16E+05 |
| 10 | 10 | DoA | B2 | 1,09E+07 | 5,48E+04 | 0,00E+00 | 7,62E+04 | 1,53E+05 | 7,73E+04 | 2,49E+04 | 2,12E+05 | 0,00E+00 | 5,32E+06 | 3,26E+06 | 1,05E+07 | 2,81E+06 | 7,63E+06 | 4,98E+06 | 1,93E+05 | 6,30E+06 | 0,00E+00 | 6,48E+03 | 7,81E+05 | 1,19E+05 | 5,73E+05 | 6,75E+04 | 5,62E+07 | 3,39E+07 | 1,42E+07 | 2,60E+06 | 0,00E+00 | 2,14E+05 | 4,23E+05 | 4,29E+07 | 2,34E+07 | 1,79E+07 | 6,62E+05 | 1,52E+06 | 3,68E+06 | 1,78E+04 | 1,79E+04 | 0,00E+00 | 5,15E+05 | 1,36E+05 | 1,82E+05 | 0,00E+00 | 2,65E+04 | 3,38E+05 | 0,00E+00 | 8,76E+03 | 4,03E+05 | 6,50E+03 | 8,60E+05 | 4,19E+05 | 3,58E+05 | 1,04E+05 | 1,29E+07 | 5,53E+06 | 1,81E+05 |
| 11 | 10 | DoA | B3 | 5,01E+06 | 2,77E+04 | 0,00E+00 | 3,32E+04 | 0,00E+00 | 1,84E+04 | 1,03E+04 | 4,36E+04 | 0,00E+00 | 2,94E+06 | 1,03E+06 | 3,75E+06 | 9,81E+05 | 2,86E+06 | 1,89E+06 | 5,54E+04 | 3,65E+06 | 0,00E+00 | 0,00E+00 | 2,67E+05 | 4,06E+04 | 3,04E+05 | 2,73E+04 | 2,34E+07 | 1,36E+06 | 6,21E+06 | 1,38E+06 | 0,00E+00 | 7,52E+04 | 1,12E+05 | 1,54E+07 | 9,31E+06 | 6,90E+06 | 4,12E+05 | 3,88E+05 | 2,20E+06 | 0,00E+00 | 0,00E+00 | 0,00E+00 | 1,47E+05 | 5,10E+04 | 9,82E+04 | 0,00E+00 | 0,00E+00 | 1,03E+05 | 0,00E+00 | 4,05E+03 | 1,21E+05 | 0,00E+00 | 2,44E+05 | 2,53E+05 | 1,22E+05 | 2,80E+04 | 5,84E+06 | 3,12E+06 | 1,20E+05 |
| 12 | 10 | DoA | B4 | 5,22E+06 | 4,18E+04 | 0,00E+00 | 4,68E+04 | 9,84E+04 | 1,72E+05 | 3,59E+04 | 2,53E+05 | 1,74E+04 | 1,58E+06 | 2,35E+06 | 4,81E+06 | 1,69E+06 | 3,45E+06 | 2,04E+06 | 2,21E+05 | 4,30E+06 | 0,00E+00 | 4,25E+03 | 5,90E+05 | 2,79E+05 | 2,02E+05 | 1,10E+05 | 1,68E+07 | 6,87E+06 | 5,22E+06 | 1,87E+06 | 4,91E+03 | 3,86E+05 | 6,78E+05 | 2,04E+07 | 9,83E+06 | 7,64E+06 | 2,78E+05 | 1,43E+06 | 2,66E+06 | 1,79E+04 | 0,00E+00 | 0,00E+00 | 6,57E+05 | 2,11E+05 | 3,38E+05 | 0,00E+00 | 3,90E+04 | 5,46E+05 | 2,00E+03 | 6,87E+03 | 6,61E+05 | 6,01E+03 | 7,86E+05 | 3,14E+05 | 3,09E+05 | 9,09E+04 | 4,79E+06 | 3,97E+06 | 6,94E+04 |
| 9 | 10 | DiE | B5 | 1,32E+07 | 0,00E+00 | 0,00E+00 | 0,00E+00 | 0,00E+00 | 7,79E+04 | 9,48E+03 | 1,17E+05 | 0,00E+00 | 7,66E+06 | 2,54E+06 | 1,42E+07 | 3,04E+06 | 1,06E+07 | 7,50E+06 | 5,34E+05 | 5,03E+06 | 0,00E+00 | 0,00E+00 | 7,80E+05 | 1,56E+05 | 7,98E+05 | 1,00E+05 | 7,13E+07 | 5,12E+07 | 1,92E+07 | 3,36E+06 | 0,00E+00 | 5,52E+05 | 1,74E+06 | 5,16E+07 | 3,48E+07 | 2,56E+07 | 6,86E+05 | 3,10E+06 | 5,11E+06 | 0,00E+00 | 0,00E+00 | 0,00E+00 | 2,03E+06 | 7,07E+04 | 1,33E+05 | 0,00E+00 | 1,36E+04 | 5,09E+05 | 0,00E+00 | 0,00E+00 | 3,97E+05 | 0,00E+00 | 8,51E+05 | 6,72E+05 | 1,14E+06 | 9,21E+04 | 1,63E+07 | 9,56E+06 | 1,32E+05 |
| 10 | 10 | DiE | B6 | 8,79E+06 | 0,00E+00 | 0,00E+00 | 0,00E+00 | 0,00E+00 | 2,76E+04 | 5,20E+03 | 3,90E+04 | 0,00E+00 | 6,65E+06 | 1,39E+06 | 9,31E+06 | 1,88E+06 | 7,08E+06 | 5,42E+06 | 2,80E+05 | 3,55E+06 | 0,00E+00 | 5,64E+03 | 4,22E+05 | 9,06E+04 | 6,62E+05 | 4,80E+04 | 4,17E+07 | 3,73E+07 | 1,58E+07 | 2,82E+06 | 0,00E+00 | 3,21E+05 | 8,66E+05 | 3,27E+07 | 2,56E+07 | 1,83E+07 | 6,54E+05 | 1,66E+06 | 4,76E+06 | 0,00E+00 | 0,00E+00 | 0,00E+00 | 1,03E+06 | 0,00E+00 | 1,98E+05 | 0,00E+00 | 1,04E+04 | 2,78E+05 | 0,00E+00 | 0,00E+00 | 2,10E+05 | 0,00E+00 | 4,61E+05 | 5,70E+05 | 5,90E+05 | 4,56E+04 | 1,44E+07 | 7,63E+06 | 1,10E+05 |
| 11 | 10 | DiE | B7 | 1,18E+07 | 1,98E+05 | 9,02E+04 | 2,14E+05 | 4,04E+05 | 2,95E+06 | 5,41E+05 | 4,75E+06 | 8,61E+04 | 4,99E+06 | 1,22E+07 | 1,69E+07 | 3,17E+06 | 1,17E+07 | 6,29E+06 | 1,68E+06 | 3,04E+06 | 9,56E+04 | 4,82E+03 | 3,04E+06 | 4,29E+06 | 5,10E+05 | 3,06E+06 | 6,47E+07 | 4,47E+07 | 1,38E+07 | 2,22E+06 | 2,19E+03 | 6,30E+06 | 5,89E+06 | 7,23E+07 | 3,01E+07 | 2,31E+07 | 5,97E+05 | 7,57E+06 | 4,50E+06 | 9,84E+04 | 6,18E+05 | 8,74E+04 | 7,64E+06 | 3,02E+06 | 1,74E+05 | 4,80E+05 | 6,96E+05 | 6,58E+06 | 0,00E+00 | 3,64E+04 | 6,50E+06 | 6,37E+03 | 5,41E+06 | 5,10E+05 | 2,54E+06 | 3,40E+05 | 1,31E+07 | 6,59E+06 | 9,51E+04 |
| 12 | 10 | DiE | B8 | 6,65E+06 | 4,29E+04 | 0,00E+00 | 4,12E+04 | 8,90E+04 | 5,21E+05 | 1,07E+05 | 7,39E+05 | 0,00E+00 | 5,04E+06 | 2,82E+06 | 6,69E+06 | 1,09E+06 | 5,10E+06 | 3,71E+06 | 4,86E+05 | 1,40E+06 | 0,00E+00 | 0,00E+00 | 6,93E+05 | 8,42E+05 | 4,64E+05 | 6,04E+05 | 3,82E+07 | 3,43E+07 | 1,18E+07 | 2,85E+06 | 0,00E+00 | 1,40E+06 | 1,58E+06 | 2,70E+07 | 1,70E+07 | 1,15E+07 | 9,84E+05 | 1,90E+06 | 6,54E+06 | 2,24E+04 | 0,00E+00 | 0,00E+00 | 1,85E+06 | 5,79E+05 | 3,72E+05 | 0,00E+00 | 1,26E+05 | 1,45E+06 | 0,00E+00 | 7,54E+03 | 1,34E+06 | 0,00E+00 | 1,15E+06 | 5,64E+05 | 9,64E+05 | 7,75E+04 | 1,09E+07 | 4,57E+06 | 7,79E+05 |
| 9 | 10 | DeC | B9 | 1,17E+07 | 0,00E+00 | 0,00E+00 | 0,00E+00 | 0,00E+00 | 5,67E+04 | 4,55E+03 | 1,00E+05 | 0,00E+00 | 8,19E+06 | 1,40E+06 | 1,05E+07 | 1,36E+06 | 8,38E+06 | 6,54E+06 | 5,73E+05 | 1,58E+06 | 0,00E+00 | 0,00E+00 | 3,42E+05 | 1,29E+05 | 7,86E+05 | 6,25E+04 | 6,73E+07 | 4,89E+07 | 1,98E+07 | 6,54E+06 | 0,00E+00 | 3,67E+05 | 1,64E+06 | 3,79E+07 | 2,90E+07 | 1,93E+07 | 1,61E+06 | 1,95E+06 | 1,44E+07 | 1,10E+04 | 0,00E+00 | 0,00E+00 | 1,53E+06 | 0,00E+00 | 9,93E+04 | 0,00E+00 | 1,14E+04 | 3,91E+05 | 0,00E+00 | 0,00E+00 | 2,17E+05 | 0,00E+00 | 3,78E+05 | 1,20E+06 | 1,73E+06 | 3,66E+04 | 1,66E+07 | 7,28E+06 | 1,49E+06 |
| 10 | 10 | DeC | B10 | 1,00E+07 | 0,00E+00 | 0,00E+00 | 0,00E+00 | 0,00E+00 | 5,17E+04 | 5,45E+03 | 7,47E+04 | 3,66E+03 | 6,79E+06 | 1,41E+06 | 9,90E+06 | 1,30E+06 | 8,04E+06 | 5,80E+06 | 5,82E+05 | 8,12E+05 | 0,00E+00 | 4,42E+03 | 3,40E+05 | 1,37E+05 | 6,77E+05 | 3,37E+04 | 5,81E+07 | 4,56E+07 | 1,61E+07 | 4,67E+06 | 0,00E+00 | 2,94E+05 | 1,73E+06 | 3,59E+07 | 2,58E+07 | 1,69E+07 | 1,45E+06 | 1,85E+06 | 7,83E+06 | 0,00E+00 | 0,00E+00 | 0,00E+00 | 1,51E+06 | 5,20E+04 | 7,58E+04 | 0,00E+00 | 3,44E+03 | 3,43E+05 | 0,00E+00 | 0,00E+00 | 1,77E+05 | 0,00E+00 | 3,60E+05 | 5,94E+05 | 1,55E+06 | 3,09E+04 | 1,56E+07 | 3,51E+06 | 1,08E+06 |
| 11 | 10 | DeC | B11 | 1,33E+07 | 8,33E+04 | 0,00E+00 | 1,17E+05 | 2,31E+05 | 2,06E+06 | 2,78E+05 | 4,77E+06 | 2,93E+04 | 6,09E+06 | 1,20E+07 | 1,72E+07 | 1,64E+06 | 1,25E+07 | 6,88E+06 | 1,74E+06 | 5,14E+05 | 4,90E+04 | 0,00E+00 | 2,80E+06 | 3,45E+06 | 6,03E+05 | 1,60E+06 | 6,98E+07 | 4,03E+07 | 1,55E+07 | 4,11E+06 | 0,00E+00 | 6,18E+06 | 6,13E+06 | 7,22E+07 | 3,12E+07 | 2,48E+07 | 1,33E+06 | 6,29E+06 | 6,57E+06 | 4,95E+04 | 4,56E+05 | 5,17E+04 | 7,81E+06 | 1,98E+06 | 2,88E+04 | 4,66E+05 | 2,77E+05 | 6,41E+06 | 0,00E+00 | 1,64E+04 | 5,13E+06 | 0,00E+00 | 4,90E+06 | 5,37E+05 | 2,10E+06 | 1,41E+05 | 1,46E+07 | 2,24E+06 | 1,03E+06 |
| 12 | 10 | DeC | B12 | 9,96E+06 | 7,83E+04 | 0,00E+00 | 1,18E+05 | 2,09E+05 | 7,19E+04 | 2,82E+04 | 1,99E+05 | 0,00E+00 | 3,72E+06 | 3,55E+06 | 1,04E+07 | 2,90E+06 | 7,81E+06 | 4,67E+06 | 2,09E+05 | 7,22E+06 | 0,00E+00 | 9,96E+03 | 8,89E+05 | 1,43E+05 | 4,93E+05 | 6,76E+04 | 4,06E+07 | 3,16E+07 | 1,22E+07 | 2,76E+06 | 0,00E+00 | 2,55E+05 | 4,24E+05 | 4,32E+07 | 2,21E+07 | 1,68E+07 | 5,46E+05 | 1,47E+06 | 3,84E+06 | 2,25E+04 | 4,43E+04 | 0,00E+00 | 5,55E+05 | 1,62E+05 | 5,55E+04 | 0,00E+00 | 2,35E+04 | 3,85E+05 | 0,00E+00 | 1,49E+04 | 4,24E+05 | 1,39E+04 | 8,82E+05 | 4,44E+05 | 4,65E+05 | 1,13E+05 | 1,14E+07 | 6,35E+06 | 1,64E+05 |
| 9 | 20 | DoA | C1 | 1,36E+07 | 0,00E+00 | 0,00E+00 | 0,00E+00 | 0,00E+00 | 9,27E+04 | 8,09E+03 | 1,12E+05 | 0,00E+00 | 7,72E+06 | 2,93E+06 | 1,53E+07 | 3,17E+06 | 1,15E+07 | 7,75E+06 | 6,22E+05 | 4,71E+06 | 0,00E+00 | 0,00E+00 | 9,15E+05 | 1,57E+05 | 8,07E+05 | 1,17E+05 | 7,32E+07 | 5,01E+07 | 1,87E+07 | 4,10E+06 | 0,00E+00 | 6,13E+05 | 2,31E+06 | 5,50E+07 | 3,47E+07 | 2,63E+07 | 7,21E+05 | 3,86E+06 | 5,76E+06 | 0,00E+00 | 0,00E+00 | 0,00E+00 | 2,34E+06 | 5,94E+04 | 2,03E+05 | 0,00E+00 | 1,90E+04 | 5,92E+05 | 0,00E+00 | 0,00E+00 | 4,84E+05 | 0,00E+00 | 1,04E+06 | 6,97E+05 | 1,34E+06 | 1,07E+05 | 1,76E+07 | 1,10E+07 | 1,42E+05 |
| 10 | 20 | DoA | C2 | 1,03E+07 | 5,41E+04 | 0,00E+00 | 7,05E+04 | 1,33E+05 | 6,66E+04 | 1,86E+04 | 1,87E+05 | 1,23E+04 | 4,00E+06 | 2,61E+06 | 8,68E+06 | 2,47E+06 | 6,34E+06 | 4,14E+06 | 1,61E+05 | 6,49E+06 | 0,00E+00 | 8,40E+03 | 6,69E+05 | 1,61E+05 | 4,78E+05 | 7,26E+04 | 4,54E+07 | 3,04E+07 | 1,13E+07 | 3,58E+06 | 0,00E+00 | 1,84E+05 | 5,06E+05 | 3,43E+07 | 1,90E+07 | 1,48E+07 | 5,43E+05 | 1,69E+06 | 4,78E+06 | 2,21E+04 | 3,55E+04 | 0,00E+00 | 4,19E+05 | 1,61E+05 | 2,06E+05 | 0,00E+00 | 2,64E+04 | 3,13E+05 | 0,00E+00 | 9,97E+03 | 3,47E+05 | 8,88E+03 | 6,96E+05 | 5,08E+05 | 3,77E+05 | 8,29E+04 | 1,06E+07 | 7,44E+06 | 1,61E+05 |
| 11 | 20 | DoA | C3 | 1,09E+07 | 5,37E+04 | 0,00E+00 | 7,55E+04 | 1,55E+05 | 5,38E+04 | 2,17E+04 | 8,89E+04 | 9,68E+03 | 5,83E+06 | 2,14E+06 | 7,84E+06 | 2,18E+06 | 6,11E+06 | 3,93E+06 | 1,18E+05 | 8,00E+06 | 2,65E+04 | 9,07E+03 | 5,49E+05 | 1,38E+05 | 6,10E+05 | 5,19E+04 | 4,68E+07 | 3,05E+07 | 1,21E+07 | 4,08E+06 | 0,00E+00 | 1,36E+05 | 3,47E+05 | 3,19E+07 | 1,82E+07 | 1,42E+07 | 8,11E+05 | 1,19E+06 | 5,73E+06 | 2,21E+04 | 0,00E+00 | 0,00E+00 | 3,04E+05 | 1,26E+05 | 1,44E+05 | 5,57E+04 | 2,18E+04 | 1,99E+05 | 0,00E+00 | 1,07E+04 | 2,53E+05 | 1,01E+04 | 5,54E+05 | 6,17E+05 | 2,82E+05 | 5,79E+04 | 1,18E+07 | 9,10E+06 | 2,30E+05 |
| **12** | **20** | **DoA** | **C4** | **1,15E+07** | **3,04E+05** | **1,90E+05** | **3,36E+05** | **5,99E+05** | **1,96E+06** | **5,65E+05** | **3,72E+06** | **1,67E+05** | **4,35E+06** | **1,03E+07** | **1,42E+07** | **5,45E+06** | **9,66E+06** | **5,34E+06** | **1,43E+06** | **7,33E+06** | **1,84E+05** | **8,67E+03** | **2,94E+06** | **3,93E+06** | **5,84E+05** | **1,90E+06** | **3,90E+07** | **3,79E+07** | **1,20E+07** | **5,12E+06** | **0,00E+00** | **4,05E+06** | **5,70E+06** | **5,93E+07** | **2,42E+07** | **1,97E+07** | **4,43E+05** | **8,18E+06** | **5,62E+06** | **1,67E+05** | **3,26E+05** | **1,07E+05** | **5,53E+06** | **2,99E+06** | **3,59E+05** | **6,51E+05** | **7,07E+05** | **5,06E+06** | **0,00E+00** | **5,62E+04** | **6,37E+06** | **5,34E+04** | **4,88E+06** | **6,01E+05** | **1,85E+06** | **5,53E+05** | **1,16E+07** | **9,32E+06** | **1,12E+05** |
| 9 | 20 | DiE | C5 | 1,27E+07 | 0,00E+00 | 0,00E+00 | 0,00E+00 | 0,00E+00 | 6,05E+04 | 8,29E+03 | 7,57E+04 | 0,00E+00 | 7,22E+06 | 2,20E+06 | 1,31E+07 | 2,81E+06 | 9,98E+06 | 7,17E+06 | 4,77E+05 | 4,53E+06 | 0,00E+00 | 0,00E+00 | 6,73E+05 | 1,21E+05 | 7,84E+05 | 8,63E+04 | 6,88E+07 | 4,67E+07 | 1,82E+07 | 4,68E+06 | 0,00E+00 | 4,50E+05 | 1,61E+06 | 4,55E+07 | 3,18E+07 | 2,36E+07 | 7,38E+05 | 2,66E+06 | 6,82E+06 | 0,00E+00 | 0,00E+00 | 0,00E+00 | 1,72E+06 | 9,41E+04 | 1,62E+05 | 0,00E+00 | 1,32E+04 | 4,38E+05 | 0,00E+00 | 0,00E+00 | 3,47E+05 | 0,00E+00 | 7,77E+05 | 8,12E+05 | 1,04E+06 | 7,95E+04 | 1,75E+07 | 1,18E+07 | 1,34E+05 |
| 10 | 20 | DiE | C6 | 1,23E+07 | 0,00E+00 | 0,00E+00 | 0,00E+00 | 0,00E+00 | 5,10E+04 | 4,03E+03 | 4,49E+04 | 0,00E+00 | 7,13E+06 | 1,86E+06 | 1,22E+07 | 2,51E+06 | 9,51E+06 | 7,02E+06 | 3,93E+05 | 4,14E+06 | 0,00E+00 | 0,00E+00 | 5,96E+05 | 8,34E+04 | 7,81E+05 | 5,26E+04 | 6,98E+07 | 4,84E+07 | 1,90E+07 | 5,28E+06 | 0,00E+00 | 3,87E+05 | 1,19E+06 | 4,30E+07 | 3,07E+07 | 2,35E+07 | 7,09E+05 | 2,12E+06 | 7,61E+06 | 0,00E+00 | 0,00E+00 | 0,00E+00 | 1,45E+06 | 0,00E+00 | 1,71E+05 | 0,00E+00 | 0,00E+00 | 3,53E+05 | 0,00E+00 | 0,00E+00 | 2,79E+05 | 0,00E+00 | 6,41E+05 | 8,53E+05 | 9,34E+05 | 6,59E+04 | 1,79E+07 | 1,27E+07 | 1,22E+05 |
| 11 | 20 | DiE | C7 | 1,37E+07 | 1,46E+05 | 0,00E+00 | 1,10E+05 | 3,07E+05 | 2,48E+06 | 4,07E+05 | 4,44E+06 | 8,07E+04 | 5,92E+06 | 1,20E+07 | 1,87E+07 | 3,03E+06 | 1,37E+07 | 7,35E+06 | 1,79E+06 | 4,31E+06 | 7,75E+04 | 0,00E+00 | 3,02E+06 | 3,38E+06 | 6,28E+05 | 2,31E+06 | 6,60E+07 | 4,82E+07 | 1,63E+07 | 3,82E+06 | 0,00E+00 | 6,42E+06 | 5,82E+06 | 7,70E+07 | 3,29E+07 | 2,64E+07 | 6,95E+05 | 6,81E+06 | 6,90E+06 | 7,13E+04 | 3,67E+05 | 6,41E+04 | 8,02E+06 | 2,18E+06 | 1,70E+05 | 2,14E+05 | 6,47E+05 | 6,18E+06 | 0,00E+00 | 2,92E+04 | 6,24E+06 | 0,00E+00 | 5,47E+06 | 7,12E+05 | 2,77E+06 | 3,18E+05 | 1,53E+07 | 1,05E+07 | 1,14E+05 |
| 12 | 20 | DiE | C8 | 1,02E+07 | 0,00E+00 | 0,00E+00 | 0,00E+00 | 0,00E+00 | 5,14E+04 | 3,15E+03 | 5,05E+04 | 0,00E+00 | 8,23E+06 | 1,07E+06 | 8,62E+06 | 1,03E+06 | 7,06E+06 | 5,87E+06 | 4,81E+05 | 1,38E+06 | 0,00E+00 | 0,00E+00 | 2,58E+05 | 1,03E+05 | 8,13E+05 | 3,00E+04 | 5,65E+07 | 5,09E+07 | 1,92E+07 | 8,00E+06 | 0,00E+00 | 2,64E+05 | 1,39E+06 | 2,94E+07 | 2,47E+07 | 1,63E+07 | 1,81E+06 | 1,56E+06 | 1,45E+07 | 0,00E+00 | 0,00E+00 | 0,00E+00 | 1,19E+06 | 0,00E+00 | 4,38E+05 | 0,00E+00 | 7,39E+03 | 2,68E+05 | 0,00E+00 | 0,00E+00 | 1,72E+05 | 0,00E+00 | 3,05E+05 | 1,08E+06 | 1,29E+06 | 2,86E+04 | 1,58E+07 | 6,36E+06 | 1,61E+06 |
| 9 | 20 | DeC | C9 | 1,05E+07 | 0,00E+00 | 0,00E+00 | 0,00E+00 | 0,00E+00 | 5,49E+04 | 4,96E+03 | 5,07E+04 | 0,00E+00 | 8,89E+06 | 1,12E+06 | 8,86E+06 | 1,26E+06 | 7,02E+06 | 6,10E+06 | 4,85E+05 | 2,02E+06 | 0,00E+00 | 2,80E+03 | 2,83E+05 | 1,09E+05 | 9,13E+05 | 4,35E+04 | 5,96E+07 | 5,63E+07 | 2,04E+07 | 6,89E+06 | 0,00E+00 | 3,06E+05 | 1,34E+06 | 3,01E+07 | 2,55E+07 | 1,67E+07 | 1,97E+06 | 1,57E+06 | 1,31E+07 | 0,00E+00 | 0,00E+00 | 0,00E+00 | 1,20E+06 | 0,00E+00 | 1,00E+05 | 0,00E+00 | 1,16E+04 | 2,98E+05 | 0,00E+00 | 0,00E+00 | 1,85E+05 | 0,00E+00 | 3,04E+05 | 9,85E+05 | 1,42E+06 | 3,24E+04 | 1,92E+07 | 8,30E+06 | 1,87E+06 |
| 10 | 20 | DeC | C10 | 8,89E+06 | 0,00E+00 | 0,00E+00 | 0,00E+00 | 0,00E+00 | 3,96E+04 | 3,52E+03 | 3,32E+04 | 0,00E+00 | 7,57E+06 | 1,08E+06 | 9,19E+06 | 9,43E+05 | 7,46E+06 | 6,12E+06 | 5,09E+05 | 5,77E+05 | 0,00E+00 | 0,00E+00 | 2,80E+05 | 6,44E+04 | 7,70E+05 | 3,46E+04 | 5,86E+07 | 4,72E+07 | 1,81E+07 | 5,55E+06 | 0,00E+00 | 2,45E+05 | 1,39E+06 | 3,18E+07 | 2,51E+07 | 1,63E+07 | 1,57E+06 | 1,38E+06 | 8,95E+06 | 0,00E+00 | 0,00E+00 | 0,00E+00 | 1,24E+06 | 0,00E+00 | 6,54E+04 | 0,00E+00 | 0,00E+00 | 2,69E+05 | 0,00E+00 | 0,00E+00 | 1,43E+05 | 0,00E+00 | 2,77E+05 | 5,77E+05 | 1,42E+06 | 2,40E+04 | 1,70E+07 | 2,67E+06 | 1,25E+06 |
| 11 | 20 | DeC | C11 | 1,31E+07 | 1,34E+05 | 0,00E+00 | 1,73E+05 | 3,17E+05 | 3,67E+06 | 5,15E+05 | 6,66E+06 | 6,53E+04 | 8,18E+06 | 1,57E+07 | 2,36E+07 | 2,67E+06 | 1,70E+07 | 9,35E+06 | 2,44E+06 | 9,78E+05 | 1,30E+05 | 4,75E+03 | 3,91E+06 | 5,08E+06 | 8,46E+05 | 2,36E+06 | 8,90E+07 | 7,00E+07 | 2,09E+07 | 8,21E+06 | 0,00E+00 | 8,82E+06 | 7,91E+06 | 9,55E+07 | 3,93E+07 | 3,32E+07 | 1,81E+06 | 8,77E+06 | 1,15E+07 | 7,54E+04 | 7,72E+05 | 8,67E+04 | 1,07E+07 | 2,99E+06 | 5,67E+04 | 6,21E+05 | 7,20E+05 | 8,97E+06 | 0,00E+00 | 3,02E+04 | 7,40E+06 | 7,25E+03 | 6,89E+06 | 1,04E+06 | 3,45E+06 | 2,62E+05 | 1,93E+07 | 4,52E+06 | 1,60E+06 |
| 12 | 20 | DeC | C12 | 1,14E+07 | 7,35E+04 | 0,00E+00 | 1,09E+05 | 2,21E+05 | 8,48E+04 | 3,27E+04 | 2,53E+05 | 1,42E+04 | 4,47E+06 | 3,30E+06 | 1,03E+07 | 2,85E+06 | 7,63E+06 | 4,69E+06 | 2,00E+05 | 7,37E+06 | 3,01E+04 | 9,03E+03 | 8,62E+05 | 1,38E+05 | 5,16E+05 | 9,83E+04 | 5,08E+07 | 3,06E+07 | 1,23E+07 | 4,05E+06 | 7,02E+03 | 2,26E+05 | 6,23E+05 | 4,15E+07 | 2,15E+07 | 1,68E+07 | 6,17E+05 | 2,05E+06 | 5,70E+06 | 2,23E+04 | 4,69E+04 | 0,00E+00 | 5,44E+05 | 2,04E+05 | 1,95E+05 | 7,75E+04 | 3,02E+04 | 4,39E+05 | 1,27E+04 | 1,23E+04 | 4,28E+05 | 1,23E+04 | 8,85E+05 | 5,78E+05 | 4,92E+05 | 1,09E+05 | 1,15E+07 | 9,15E+06 | 2,06E+05 |
| 9 | 30 | DoA | D1 | 1,37E+07 | 0,00E+00 | 0,00E+00 | 0,00E+00 | 0,00E+00 | 1,27E+05 | 1,33E+04 | 1,84E+05 | 1,15E+04 | 9,09E+06 | 3,32E+06 | 1,93E+07 | 4,31E+06 | 1,45E+07 | 9,98E+06 | 6,76E+05 | 6,50E+06 | 0,00E+00 | 2,77E+03 | 1,03E+06 | 2,83E+05 | 1,08E+06 | 1,54E+05 | 7,18E+07 | 5,25E+07 | 2,43E+07 | 6,96E+06 | 0,00E+00 | 6,96E+05 | 3,39E+06 | 6,64E+07 | 4,18E+07 | 2,73E+07 | 9,40E+05 | 6,11E+06 | 9,16E+06 | 0,00E+00 | 0,00E+00 | 0,00E+00 | 2,51E+06 | 9,16E+04 | 2,43E+05 | 0,00E+00 | 2,88E+04 | 8,70E+05 | 0,00E+00 | 0,00E+00 | 5,48E+05 | 0,00E+00 | 1,23E+06 | 1,04E+06 | 1,66E+06 | 1,20E+05 | 2,29E+07 | 1,66E+07 | 1,60E+05 |
| 10 | 30 | DoA | D2 | 1,15E+07 | 5,10E+04 | 4,00E+04 | 6,00E+04 | 1,31E+05 | 8,07E+04 | 2,94E+04 | 2,65E+05 | 1,31E+04 | 5,31E+06 | 2,94E+06 | 8,94E+06 | 2,42E+06 | 6,83E+06 | 4,31E+06 | 2,28E+05 | 5,16E+06 | 0,00E+00 | 6,01E+03 | 6,84E+05 | 1,84E+05 | 5,20E+05 | 9,89E+04 | 4,91E+07 | 3,31E+07 | 1,25E+07 | 3,69E+06 | 0,00E+00 | 2,29E+05 | 6,89E+05 | 3,61E+07 | 1,98E+07 | 1,55E+07 | 5,25E+05 | 2,19E+06 | 4,44E+06 | 1,73E+04 | 2,77E+04 | 5,37E+03 | 5,33E+05 | 1,87E+05 | 1,99E+05 | 0,00E+00 | 2,42E+04 | 3,86E+05 | 0,00E+00 | 1,03E+04 | 4,27E+05 | 9,26E+03 | 7,56E+05 | 4,32E+05 | 3,39E+05 | 1,14E+05 | 1,19E+07 | 6,79E+06 | 1,32E+05 |
| 11 | 30 | DoA | D3 | 8,46E+06 | 4,15E+04 | 0,00E+00 | 4,35E+04 | 9,11E+04 | 3,14E+04 | 1,81E+04 | 7,61E+04 | 7,16E+03 | 5,30E+06 | 1,01E+06 | 4,43E+06 | 1,08E+06 | 3,81E+06 | 2,39E+06 | 7,90E+04 | 4,98E+06 | 0,00E+00 | 5,14E+03 | 2,65E+05 | 7,17E+04 | 4,69E+05 | 3,64E+04 | 3,11E+07 | 1,96E+06 | 8,55E+06 | 2,73E+06 | 0,00E+00 | 9,87E+04 | 1,72E+05 | 1,87E+07 | 1,14E+07 | 8,99E+06 | 7,73E+05 | 6,18E+05 | 4,27E+06 | 1,72E+04 | 0,00E+00 | 0,00E+00 | 1,70E+05 | 7,18E+04 | 1,55E+05 | 0,00E+00 | 1,28E+04 | 1,24E+05 | -6,66E+02 | 0,00E+00 | 1,56E+05 | 9,03E+03 | 2,65E+05 | 4,22E+05 | 1,66E+05 | 3,57E+04 | 8,44E+06 | 6,45E+06 | 2,48E+05 |
| 12 | 30 | DoA | D4 | 1,34E+07 | 1,93E+05 | 1,16E+05 | 1,84E+05 | 3,56E+05 | 9,93E+05 | 3,33E+05 | 2,91E+06 | 1,16E+05 | 6,79E+06 | 8,77E+06 | 1,72E+07 | 4,27E+06 | 1,36E+07 | 7,34E+06 | 1,36E+06 | 6,49E+06 | 9,75E+04 | 8,78E+03 | 2,34E+06 | 2,07E+06 | 7,20E+05 | 9,78E+05 | 7,34E+07 | 4,47E+07 | 1,74E+07 | 4,90E+06 | 0,00E+00 | 2,49E+06 | 4,91E+06 | 7,00E+07 | 3,16E+07 | 2,57E+07 | 5,91E+05 | 7,47E+06 | 5,41E+06 | 9,78E+04 | 2,48E+05 | 5,03E+04 | 4,56E+06 | 1,69E+06 | 2,50E+05 | 3,02E+05 | 3,51E+05 | 3,56E+06 | 0,00E+00 | 3,48E+04 | 4,14E+06 | 3,90E+04 | 3,60E+06 | 5,33E+05 | 1,82E+06 | 3,99E+05 | 1,68E+07 | 8,69E+06 | 2,01E+05 |
| 9 | 30 | DiE | D5 | 1,37E+07 | 0,00E+00 | 0,00E+00 | 0,00E+00 | 0,00E+00 | 1,37E+05 | 1,55E+04 | 3,46E+05 | 1,30E+04 | 6,83E+06 | 2,89E+06 | 1,38E+07 | 3,38E+06 | 1,10E+07 | 6,71E+06 | 8,86E+05 | 6,21E+06 | 0,00E+00 | 0,00E+00 | 9,04E+05 | 3,19E+05 | 6,75E+05 | 1,80E+05 | 6,59E+07 | 4,99E+07 | 1,55E+07 | 5,55E+06 | 0,00E+00 | 8,90E+05 | 3,48E+06 | 5,22E+07 | 2,98E+07 | 2,32E+07 | 6,10E+05 | 6,00E+06 | 7,04E+06 | 0,00E+00 | 0,00E+00 | 0,00E+00 | 3,14E+06 | 1,21E+05 | 2,52E+05 | 0,00E+00 | 2,82E+04 | 8,78E+05 | 0,00E+00 | 0,00E+00 | 7,00E+05 | 0,00E+00 | 1,09E+06 | 7,91E+05 | 1,60E+06 | 1,55E+05 | 1,56E+07 | 1,45E+07 | 1,20E+05 |
| 10 | 30 | DiE | D6 | 1,29E+07 | 0,00E+00 | 0,00E+00 | 0,00E+00 | 0,00E+00 | 4,84E+04 | 8,56E+03 | 7,94E+04 | 1,03E+04 | 7,40E+06 | 1,83E+06 | 1,20E+07 | 2,48E+06 | 9,36E+06 | 6,43E+06 | 5,34E+05 | 4,68E+06 | 0,00E+00 | 2,81E+03 | 6,12E+05 | 1,29E+05 | 7,20E+05 | 6,92E+04 | 6,54E+07 | 3,88E+07 | 1,64E+07 | 5,42E+06 | 0,00E+00 | 4,66E+05 | 1,92E+06 | 4,07E+07 | 2,88E+07 | 2,21E+07 | 6,60E+05 | 3,44E+06 | 7,69E+06 | 0,00E+00 | 0,00E+00 | 0,00E+00 | 1,81E+06 | 4,14E+04 | 1,94E+05 | 0,00E+00 | 1,24E+04 | 4,38E+05 | 0,00E+00 | 0,00E+00 | 3,49E+05 | 0,00E+00 | 6,61E+05 | 8,42E+05 | 1,04E+06 | 8,34E+04 | 1,65E+07 | 1,38E+07 | 1,23E+05 |
| 11 | 30 | DiE | D7 | 1,11E+07 | 7,17E+04 | 0,00E+00 | 5,97E+04 | 1,49E+05 | 1,39E+06 | 2,77E+05 | 3,53E+06 | 7,40E+04 | 4,12E+06 | 7,29E+06 | 1,25E+07 | 2,34E+06 | 8,82E+06 | 4,89E+06 | 1,37E+06 | 3,51E+06 | 4,79E+04 | 2,92E+03 | 1,89E+06 | 2,65E+06 | 3,98E+05 | 1,86E+06 | 5,01E+07 | 3,01E+07 | 1,09E+07 | 4,68E+06 | 0,00E+00 | 4,10E+06 | 5,78E+06 | 5,09E+07 | 2,25E+07 | 1,85E+07 | 6,26E+05 | 7,63E+06 | 8,31E+06 | 4,57E+04 | 2,63E+05 | 4,77E+04 | 5,49E+06 | 1,79E+06 | 1,39E+05 | 1,94E+05 | 4,04E+05 | 4,45E+06 | 0,00E+00 | 1,79E+04 | 3,95E+06 | 0,00E+00 | 3,41E+06 | 7,67E+05 | 1,84E+06 | 2,54E+05 | 1,12E+07 | 1,11E+07 | 1,19E+05 |
| 12 | 30 | DiE | D8 | 1,28E+07 | 0,00E+00 | 0,00E+00 | 0,00E+00 | 0,00E+00 | 1,23E+05 | 1,14E+04 | 2,69E+05 | 0,00E+00 | 1,03E+07 | 2,33E+06 | 1,62E+07 | 2,17E+06 | 1,30E+07 | 8,89E+06 | 1,24E+06 | 2,82E+06 | 0,00E+00 | 4,79E+03 | 6,16E+05 | 2,99E+05 | 8,50E+05 | 1,09E+05 | 6,63E+07 | 6,63E+07 | 2,30E+07 | 1,07E+07 | 0,00E+00 | 7,55E+05 | 4,43E+06 | 5,67E+07 | 3,72E+07 | 2,73E+07 | 2,45E+06 | 4,95E+06 | 2,19E+07 | 0,00E+00 | 0,00E+00 | 0,00E+00 | 3,29E+06 | 9,73E+04 | 2,27E+05 | 0,00E+00 | 1,76E+04 | 8,16E+05 | 1,44E+03 | 0,00E+00 | 4,80E+05 | 0,00E+00 | 6,84E+05 | 1,23E+06 | 2,70E+06 | 7,69E+04 | 2,29E+07 | 1,31E+07 | 2,35E+06 |
| 9 | 30 | DeC | D9 | 1,24E+07 | 0,00E+00 | 0,00E+00 | 0,00E+00 | 0,00E+00 | 5,05E+04 | 4,29E+03 | 5,18E+04 | 0,00E+00 | 8,13E+06 | 9,25E+05 | 8,79E+06 | 1,12E+06 | 7,42E+06 | 6,05E+06 | 5,54E+05 | 1,56E+06 | 0,00E+00 | 5,85E+03 | 2,40E+05 | 1,27E+05 | 7,55E+05 | 4,71E+04 | 5,99E+07 | 5,60E+07 | 1,80E+07 | 9,26E+06 | 0,00E+00 | 3,12E+05 | 1,86E+06 | 3,00E+07 | 2,48E+07 | 1,68E+07 | 1,88E+06 | 2,12E+06 | 1,74E+07 | 0,00E+00 | 0,00E+00 | 0,00E+00 | 1,33E+06 | 3,96E+04 | 2,04E+05 | 0,00E+00 | 6,10E+03 | 3,09E+05 | 0,00E+00 | 0,00E+00 | 1,88E+05 | 0,00E+00 | 2,73E+05 | 1,08E+06 | 1,44E+06 | 3,29E+04 | 1,77E+07 | 7,36E+06 | 1,77E+06 |
| 10 | 30 | DeC | D10 | 8,86E+06 | 0,00E+00 | 0,00E+00 | 0,00E+00 | 0,00E+00 | 3,20E+04 | 3,82E+03 | 6,30E+04 | 0,00E+00 | 7,57E+06 | 7,37E+05 | 7,68E+06 | 7,70E+05 | 6,46E+06 | 5,09E+06 | 5,27E+05 | 5,69E+05 | 0,00E+00 | 4,06E+03 | 1,99E+05 | 6,55E+04 | 6,78E+05 | 3,39E+04 | 4,96E+07 | 3,23E+07 | 1,57E+07 | 6,64E+06 | 0,00E+00 | 2,32E+05 | 1,71E+06 | 2,62E+07 | 2,04E+07 | 1,35E+07 | 1,61E+06 | 1,71E+06 | 1,04E+07 | 0,00E+00 | 0,00E+00 | 0,00E+00 | 1,20E+06 | 0,00E+00 | 1,31E+05 | 0,00E+00 | 0,00E+00 | 2,34E+05 | 0,00E+00 | 0,00E+00 | 1,34E+05 | 0,00E+00 | 2,00E+05 | 5,98E+05 | 1,21E+06 | 2,34E+04 | 1,52E+07 | 2,79E+06 | 1,31E+06 |
| 11 | 30 | DeC | D11 | 1,30E+07 | 1,15E+05 | 4,55E+04 | 1,43E+05 | 2,27E+05 | 3,46E+06 | 6,20E+05 | 8,58E+06 | 5,44E+04 | 8,23E+06 | 1,31E+07 | 2,16E+07 | 2,50E+06 | 1,63E+07 | 8,61E+06 | 2,61E+06 | 9,93E+05 | 1,02E+05 | 4,07E+03 | 3,51E+06 | 6,90E+06 | 7,32E+05 | 3,69E+06 | 8,49E+07 | 5,99E+07 | 1,86E+07 | 1,20E+07 | 0,00E+00 | 9,62E+06 | 1,03E+07 | 8,59E+07 | 3,60E+07 | 3,06E+07 | 1,92E+06 | 1,28E+07 | 1,67E+07 | 8,34E+04 | 9,57E+05 | 9,63E+04 | 1,12E+07 | 3,74E+06 | 9,09E+04 | 7,66E+05 | 6,50E+05 | 1,04E+07 | 0,00E+00 | 2,26E+04 | 8,51E+06 | 7,91E+03 | 6,14E+06 | 1,01E+06 | 3,26E+06 | 2,68E+05 | 1,90E+07 | 4,53E+06 | 1,58E+06 |
| 12 | 30 | DeC | D12 | 1,41E+07 | 9,00E+04 | 6,95E+04 | 1,09E+05 | 2,04E+05 | 1,09E+05 | 4,58E+04 | 4,36E+05 | 1,42E+04 | 5,23E+06 | 3,46E+06 | 1,09E+07 | 3,06E+06 | 8,67E+06 | 5,16E+06 | 2,89E+05 | 6,24E+06 | 3,04E+04 | 6,62E+03 | 8,61E+05 | 2,29E+05 | 5,29E+05 | 1,09E+05 | 5,66E+07 | 3,58E+07 | 1,38E+07 | 4,31E+06 | 0,00E+00 | 2,87E+05 | 7,60E+05 | 4,53E+07 | 2,33E+07 | 1,88E+07 | 5,24E+05 | 2,55E+06 | 5,34E+06 | 3,17E+04 | 6,23E+04 | 1,07E+04 | 7,21E+05 | 1,94E+05 | 3,56E+05 | 7,32E+04 | 4,04E+04 | 4,89E+05 | 6,08E+03 | 1,37E+04 | 5,86E+05 | 1,24E+04 | 9,44E+05 | 4,94E+05 | 5,00E+05 | 1,54E+05 | 1,35E+07 | 8,21E+06 | 1,45E+05 |

Table S1: Full factorial response data analyzed in the laboratory. The correspondances among numbers and analytes are (1) 25I–NBOMe (2) 2C-B (3) 2C-H (4) 2C-T-4 (5) 2C-T-7 (6) 2-FMC (7) 2-MeOMet-Cathinone (8) 4-MethEt-Cathinone (9) 6-MAM (10) AB005 (11) Acetylfentanyl (12) Acrylfentanyl (13) Alfentanyl (14) α-Methyl-fentanyl (15) α-Methyl-thiofentanyl (16) α-PVP (17) Alprazolam (18) Amphetamine (19) Benzoylecgonine (20) β-hydroxy-fentanyl (21) Buphedrone (22) Buprenorphine (23) Butylone (24) Butyrylfentanyl (25) (±)cis-3-methyl-fentanyl (26) (±)cis-3-methyl-thiofentanyl (27) Clonazepam (28) Codeine (29) Cocaine (30) Diethylproprion (31) Fentanyl (32) Fluorofentanyl (33) Furanylfentanyl (34) JWH200 (35) Ketamine (36) Lormetazepam (37) MDA (38) MDEA (39) MDMA (40) MDPV (41) Mephedrone (42) Methadone (43) Methamphetamine (44) Methylone (45) Methoxetamine (46) Morphine (47) Norbuprenorphine (48) Norcocaine (49) Norfentanyl (50) Ocfentanyl (51) Oxazepam (52) Phencyclidine (53) Remifentanyl (54) Sufentanyl (55) Triazolam (56) WIN-55,212
